# Supplementary material for: The divisome but not the elongasome organizes capsule synthesis in Streptococcus pneumoniae
Source: Nat Commun. 2023 Jun 1;14:3170. doi: 10.1038/s41467-023-38904-9 (PMC10233549; doi:10.1038/s41467-023-38904-9)
Supplement: Supplementary file 1 — Supplementary Information [file 41467_2023_38904_MOESM1_ESM.pdf]

SUPPLEMENTAL INFORMATION

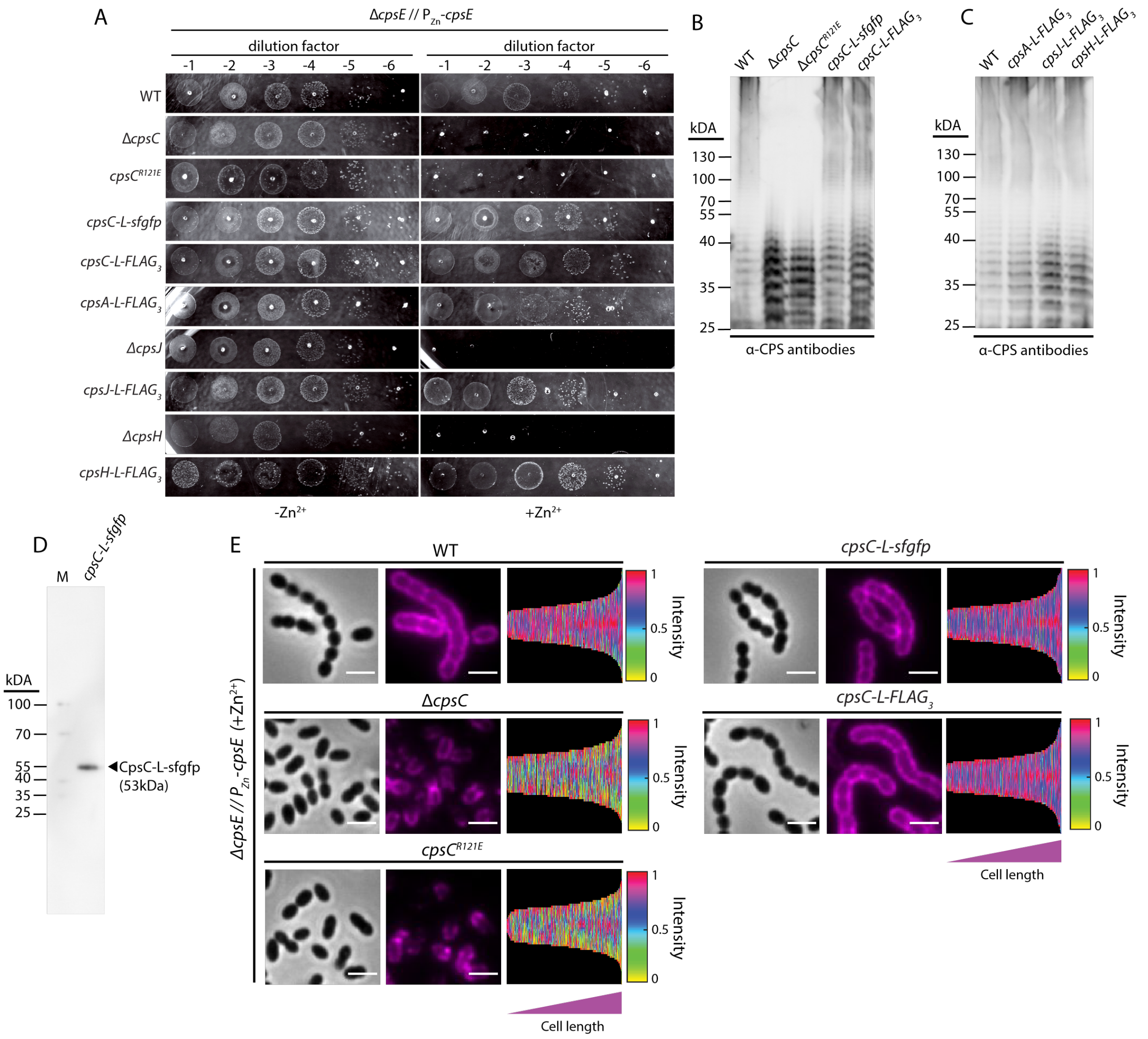

Supplemental Fig. S1. $FLAG_3$ and sfGFP tagged CpsC, CpsA, CpsJ, CpsH, and CpsA constructs are functional. (A) Strains NUS0267 [Δ*cpsE* // P_{Zn}-*cpsE*], NUS0679 [*rpsL1* Δ*cpsC* Δ*cpsE* // Δ*bgaA*::P_{Zn}-*cpsE*], NUS1174 [*rpsL1* *cpsC*^{R121E} Δ*cpsE* // Δ*bgaA*::P_{Zn}-*cpsE*], NUS0882 [*cpsC-L-sfgfp* Δ*cpsE* // P_{Zn}-*cpsE*], NUS1090 [*rpsL1 cpsC-L-FLAG*$_3$ Δ*cpsE* // Δ*bgaA*::P_{Zn}-*cpsE*], NUS0546 [*rpsL1* Δ*cpsA*::P-*sacB-kan-rpsL*$^+$ Δ*cpsE* // Δ*bgaA*::P_{Zn}-*cpsE*], NUS0734 [*rpsL1 cpsA-L-FLAG*$_3$ Δ*cpsE* // Δ*bgaA*::P_{Zn}-*cpsE*], NUS0713 [*rpsL1* Δ*cpsE* Δ*cpsJ*::P-*sacB-kan-rpsL*$^+$ // Δ*bgaA*::P_{Zn}-*cpsE*], NUS03063 [*rpsL1* Δ*cpsE cpsJ-L-FLAG*$_3$ // Δ*bgaA*::P_{Zn}-*cpsE*], NUS1137 [*rpsL1* Δ*cpsE* Δ*cpsH*::P-*sacB-kan-rpsL*$^+$ // Δ*bgaA*::P_{Zn}-*cpsE*] and NUS3064 [*rpsL1 cpsH-L-FLAG*$_3$ Δ*cpsE* // Δ*bgaA*::P_{Zn}-*cpsE*] were grown in BHI broth at 37°C in 5% CO_2 to the mid-log phase, normalized to OD_{600} of 0.2, and serially diluted. The diluted cultures were spotted on TSA plates supplemented with Zn^{2+} to induce capsule production. Plates were imaged after incubation at 37°C in 5% CO_2 overnight. Experiments were performed independently twice with similar

results. (B) Strains NUS0267, NUS00679, NUS1174, NUS0882, NUS1090 (C) NUS0267, NUS0734, NUS0363 and NUS3064 were grown in BHI supplemented with Zn^{2+} for 1 hour. Cells were collected and the CPS in each fraction was resolved by SDS-PAGE and detected by immunoblotting as described in Methods. Experiments were performed independently twice with similar results. (D) CpsC-L-sfGFP is stable. Strain NUS0882 was grown to the mid-log phase. Cells were collected, lysed, and immunoblotted with anti-GFP antibodies as described in Methods. Experiments were performed independently twice with similar results. (E) Strains NUS0267, NUS00679, NUS1174, NUS0882, NUS1090 were grown in BHI supplemented with Zn^{2+} for 2 hours. Cells were heat-killed and visualized by immunostaining using anti-capsule antibodies and fluorescence microscopy. Shown are the representative images (left) and the associated demographs (right). Bar, 2μm. Demograph analysis includes data from three biologically independent experiments (*n*=500).

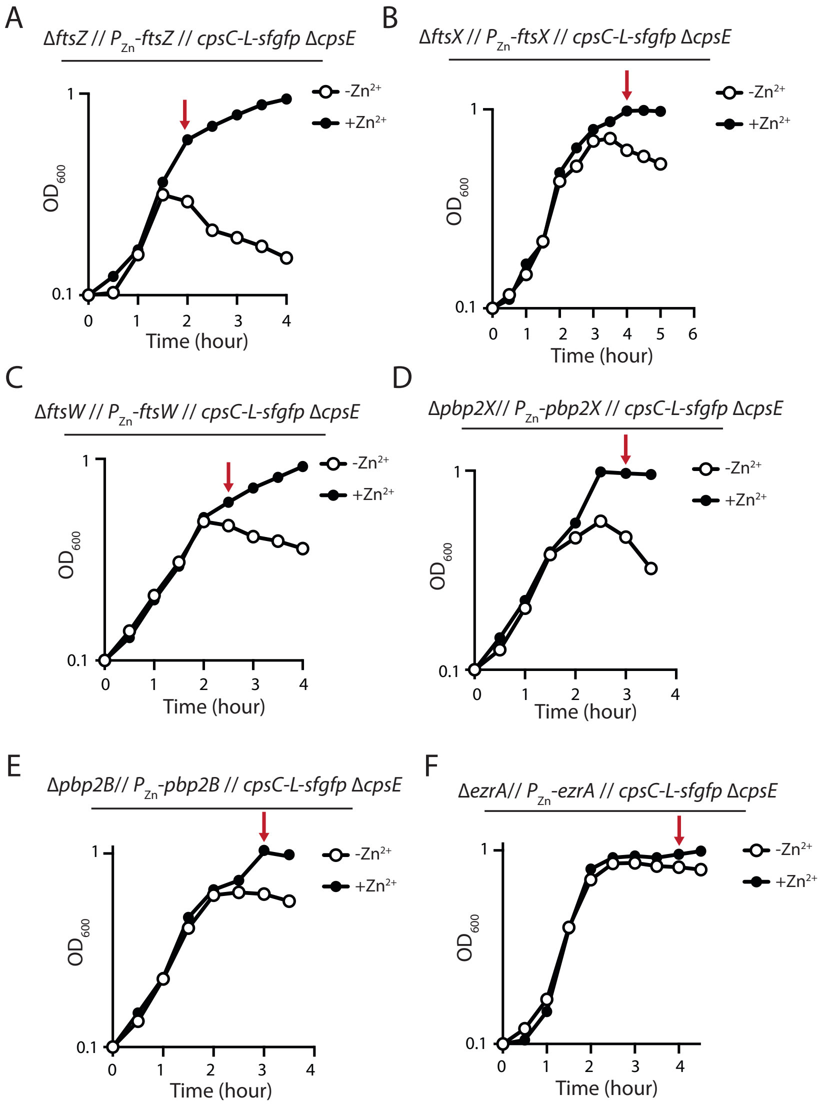

Supplemental Fig. S2. Depletion of elongasome and divisome proteins. Strains harboring CpsC-L-sfGFP with the indicated depletion cassettes were grown in BHI broth supplemented with Zn^{2+} at 37°C in 5% CO_2 (i.e., NUS1659 [Δ*ftsZ* // P_{Zn}-*ftsZ*] (A); NUS1749 [Δ*ftsX* // P_{Zn}-*ftsX*] (B); NUS1826 [Δ*ftsW* // P_{Zn}-*ftsW*] (C); NUS1751 [Δ*pbp2x* // P_{Zn}-*pbp2x*] (D); NUS1775 [Δ*pbp2b* // P_{Zn}-*pbp2b*] (E); NUS1868 [Δ*ezrA* // P_{Zn}-*ezrA*] (F).). The overnight cultures were washed with BHI without Zn^{2+} and diluted in the same medium. Growth was monitored by measuring OD_{600} over time. The arrows indicate the timing at which the cultures were collected for immunostaining.

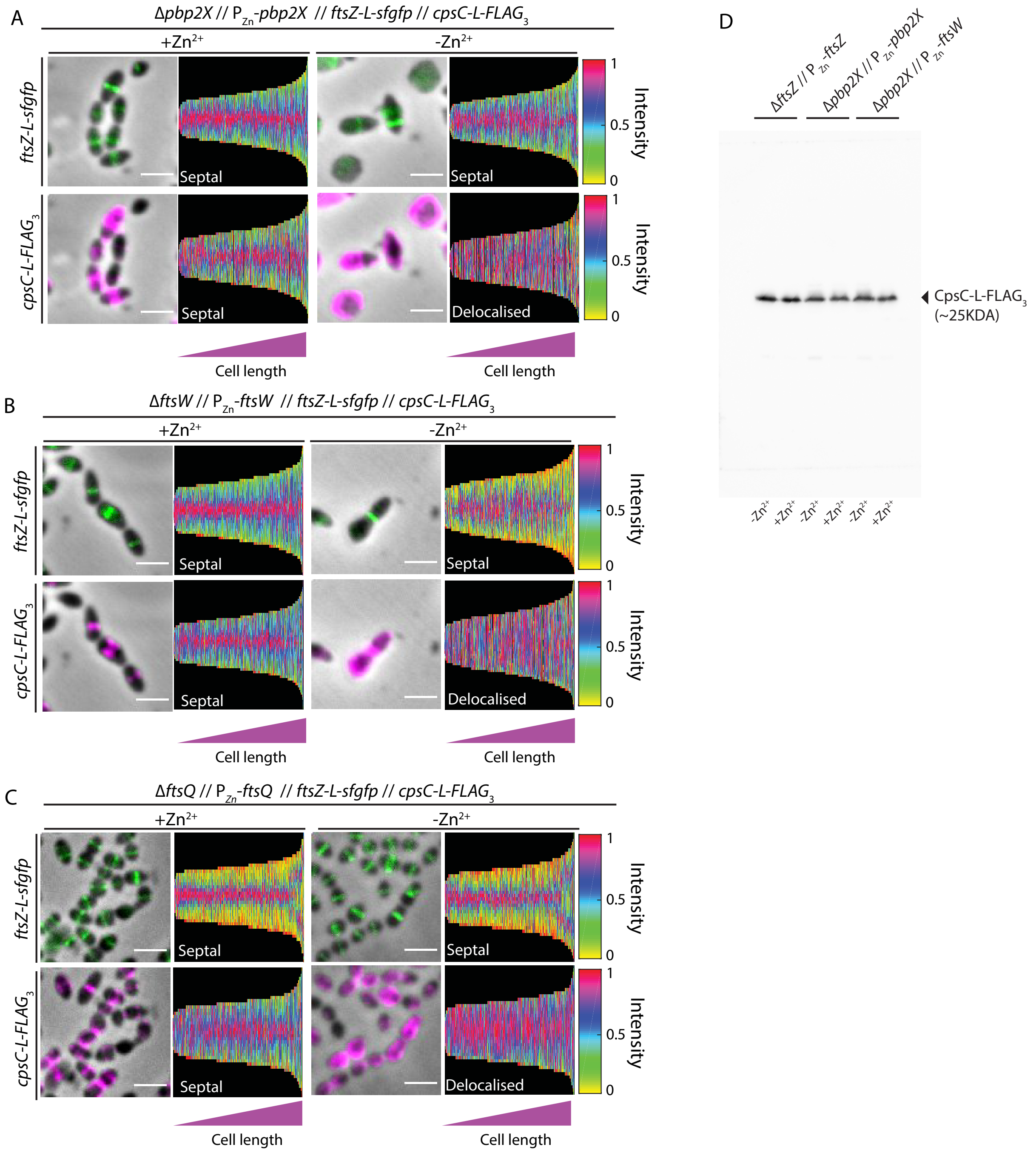

Supplemental Fig. S3. Depletion of *pbp2x* (A), *ftsW* (B), or *ftsQ* (C) delocalizes CpsC-L-FLAG3 but not FtsZ-L-sfGFP. Strains (A) NUS2185 [*cpsC-L-FLAG*$_3$ *ftsZ-L-sfgfp* Δ*pbp2X::*P-*kan-rpsL*$^+$ // *bgaA*::P$_{Zn}$-*pbp2x*], (B) NUS2184 [*cpsC-L-FLAG*$_3$ *ftsZ-L-sfgfp* Δ*ftsW*::P-*kan-rpsL*$^+$ // *bgaA*::P$_{Zn}$-*ftsW*], and (C) NUS2068 [*cpsC-L-FLAG*$_3$ *ftsZ-L-sfgfp* Δ*ftsQ*::P-*kan-rpsL*$^+$ // *bgaA*::P$_{Zn}$-*ftsQ*] were grown with the indicated proteins depleted as described in the legends of Supplemental Fig. S3. FLAG was detected by immunostaining using anti-FLAG antibodies. Cells were then imaged under fluorescence microscopy. Shown are the representative images (left) and the associated demographs (right). Bar, 2μm. Demograph analysis includes data from three biologically independent experiments (*n*=500). (D)

Strain NUS1640, NUS2185, and NUS2184 were grown with the indicated proteins depleted as described in the legends of Supplemental Fig. S3. Cells were collected, lysed, and immunoblotted with anti-FLAG antibodies as described in Methods. Experiments were performed independently thrice with similar results.

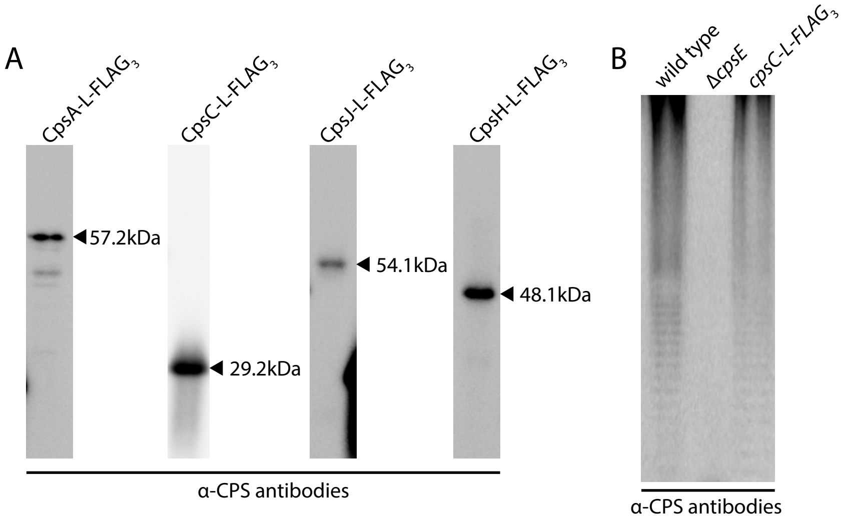

Supplemental Fig. S4. Immunoblotting demonstrates the stability of recombinant proteins and normal capsule production. (A) CpsA-L-FLAG$_3$, CpsC-L-FLAG$_3$, CpsJ-L-FLAG$_3$, and CpsH-L-FLAG$_3$ are stable. Strains NUS0734 [*cpsA-L-FLAG$_3$ ΔcpsE* // P$_{Zn}$-*cpsE*], NUS1090 [*cpsC-L-FLAG$_3$ ΔcpsE* // P$_{Zn}$-*cpsE*], NUS3063 [*cpsJ-L-FLAG$_3$ ΔcpsE* // P$_{Zn}$-*cpsE*], and NUS3064 [*cpsH-L-FLAG$_3$ ΔcpsE* // P$_{Zn}$-*cpsE*] were grown until OD$_{600}$ reached 0.2. Cultures were normalized according to their OD$_{600}$ and centrifuged to harvest the cells. Bacteria were then lysed by mutanolysin and lysozyme before mixing with the sample buffer. Proteins released were then separated by SDS-PAGE and detected by immunoblotting with anti-FLAG antibodies. Experiments were performed independently twice with similar results. (B) Strains IU1781 (wild type; the isogenic parent), HMS0001 [Δ*cpsE*], and NUS1571 [*cpsC-L-FLAG$_3$*] were grown to the mid-log phase before harvesting the cells for immunoblotting using anti-CPS antibodies. The same laddering pattern observed in the wild type and the CpsC-L-FLAG$_3$ strains indicates normal capsule production. Experiments were performed independently twice with similar results.

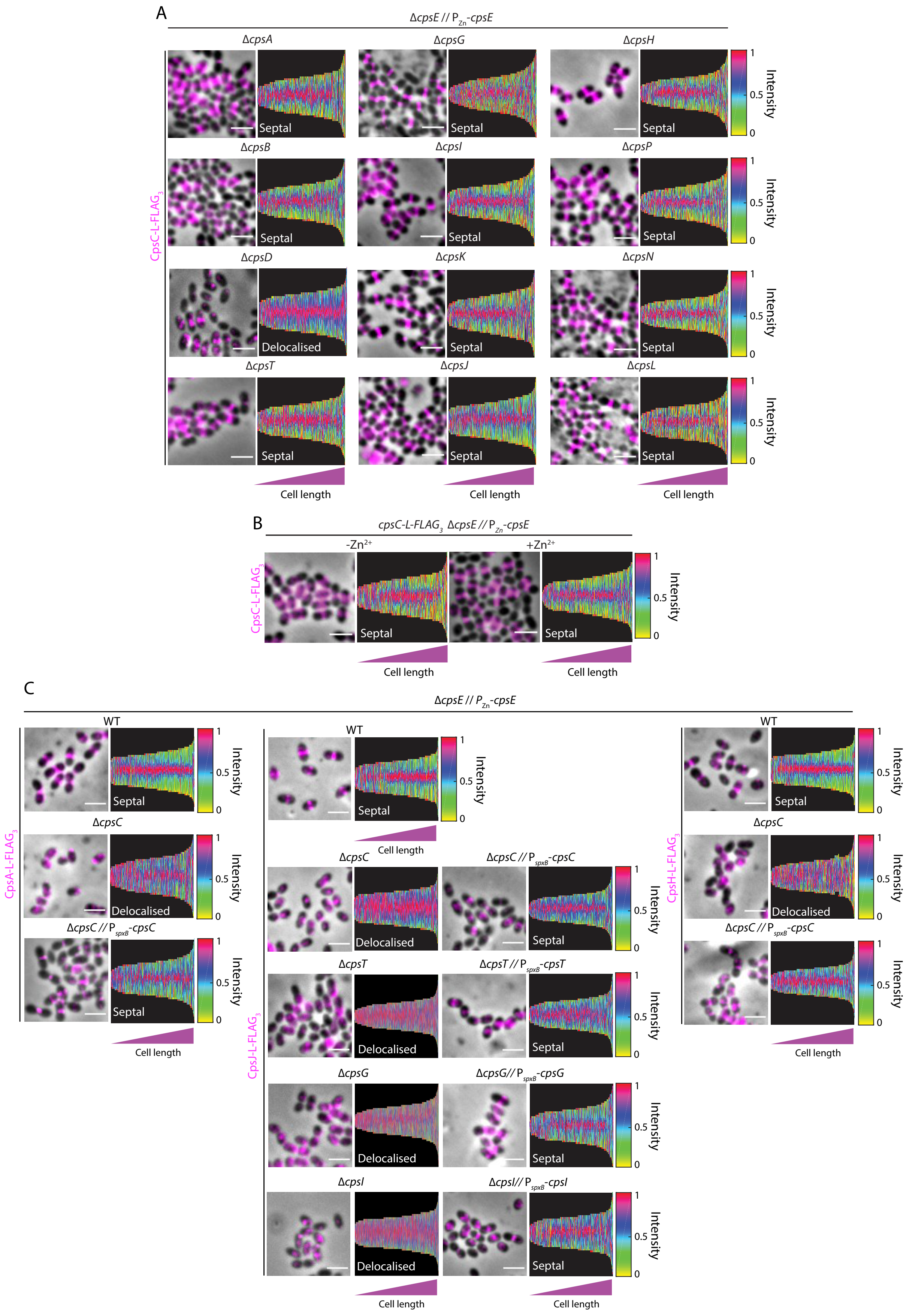
A
ΔcpsE // P_Zn-cpsE
ΔcpsA
ΔcpsG
ΔcpsH
ΔcpsB
ΔcpsI
ΔcpsP
ΔcpsD
ΔcpsK
ΔcpsN
ΔcpsT
ΔcpsJ
ΔcpsL
CpsC-L-FLAG3
Septal
Delocalised
Intensity
1
0.5
0
Cell length
B
cpsC-L-FLAG3 ΔcpsE // P_Zn-cpsE
-Zn2+
+Zn2+
CpsC-L-FLAG3
Septal
Intensity
Cell length
C
ΔcpsE // P_Zn-cpsE
WT
ΔcpsC
ΔcpsC // P_spxB-cpsC
CpsA-L-FLAG3
ΔcpsT
ΔcpsT // P_spxB-cpsT
ΔcpsG
ΔcpsG// P_spxB-cpsG
ΔcpsI
ΔcpsI// P_spxB-cpsI
CpsJ-L-FLAG3
CpsH-L-FLAG3
Septal
Delocalised
Intensity
Cell length

Supplemental Fig. S5. CpsC is upstream of other CPS proteins in the recruitment cascade. (A) Recruitment of CpsC is independent of other CPS proteins. Strain NUS1090 [*cpsC-L-FLAG*$_3$ Δ*cpsE* // P$_{Zn}$-*cpsE*] harboring the indicated deletion was grown until OD$_{600}$ is between 0.2 and 0.4. Cells were collected, immunostained, and visualized under fluorescence microscopy. Demographs were generated to get an overview of the relative location of CpsC-L-FLAG$_3$. Bar, 2μm. Shown are the representative images (left) and the associated demographs (right). Demograph analysis includes data from three biologically independent experiments (*n*=500). (B) Δ*cpsE* did not affect the location of CpsC-L-sfGFP. Strain NUS1090 was grown to the mid-log phase. Zn^{2+} was added to induce *cpsE* expression and the localization of CpsC-L-sfGFP was determined by fluorescence microscopy. Shown are the representative images (left) and the associated demographs (right). Bar, 2μm. (C) Inactivation of *cpsC* disperses CpsA-L-FLAG$_3$, CpsJ-L-FLAG$_3$, and CpsH-L-FLAG$_3$ signals. Complementation of *cpsC* restores the septal localization of CpsA, CpsJ, and CpsH. Moreoever, complementation of *cpsT*, *cpsG* and *cpsI* restores the septal localization of CpsJ. Immunostaining of the FLAG-tagged fusion proteins was done as described in (A). Strains included were NUS0734 [*cpsA-L-FLAG*$_3$ Δ*cpsE* // P$_{Zn}$-*cpsE*], NUS3100 [*cpsA-L-FLAG*$_3$ Δ*cpsC* Δ*cpsE* // P$_{Zn}$-*cpsE*], NUS3063 [*cpsJ-L-FLAG*$_3$ Δ*cpsE* // P$_{Zn}$-*cpsE*], NUS3065 [*cpsJ-L-FLAG*$_3$ Δ*cpsC* Δ*cpsE* // P$_{Zn}$-*cpsE*], NUS3064 [*cpsH-L-FLAG*$_3$ Δ*cpsE* // P$_{Zn}$-*cpsE*], NUS3103 [*cpsH-L-FLAG*$_3$ Δ*cpsC* Δ*cpsE* // P$_{Zn}$-*cpsE*], NUS3875 [*rpsL1 cpsJ-L-FLAG*$_3$ Δ*cpsC* Δ*cpsE* // Δ*bgaA*::P$_{spxB}$-*cpsC*-P-*spec*], NUS3876 [*rpsL1 cpsA-L-FLAG*$_3$ Δ*cpsC* Δ*cpsE* // Δ*bgaA*::P$_{spxB}$-*cpsC*-P-*spec*], NUS3877 [*rpsL1 cpsH-L-FLAG*$_3$ Δ*cpsC* Δ*cpsE* // Δ*bgaA*::P$_{spxB}$-*cpsC*-P-*spec*], NUS3878 [*rpsL1* Δ*cpsT*::P-*sacB-kan-rpsL*$^+$ *cpsJ-L-FLAG*$_3$ Δ*cpsE* // Δ*bgaA*::P$_{spxB}$-*cpsT*-P-*spec*], NUS3879 [*rpsL1* Δ*cpsI*:: P-*erm cpsJ-L-FLAG*$_3$ Δ*cpsE* // Δ*bgaA*::P$_{spxB}$-*cpsI*-P-*spec*] and NUS3894 [*rpsL1* Δ*cpsG*::P-*erm cpsJ-L-FLAG*$_3$ Δ*cpsE* // Δ*bgaA*::P$_{spxB}$-*cpsG*-P-*spec*] . Shown are the representative images (left) and the associated demographs (right). Demograph analysis includes data from three biologically independent experiments (*n*=500). Bar, 2μm.

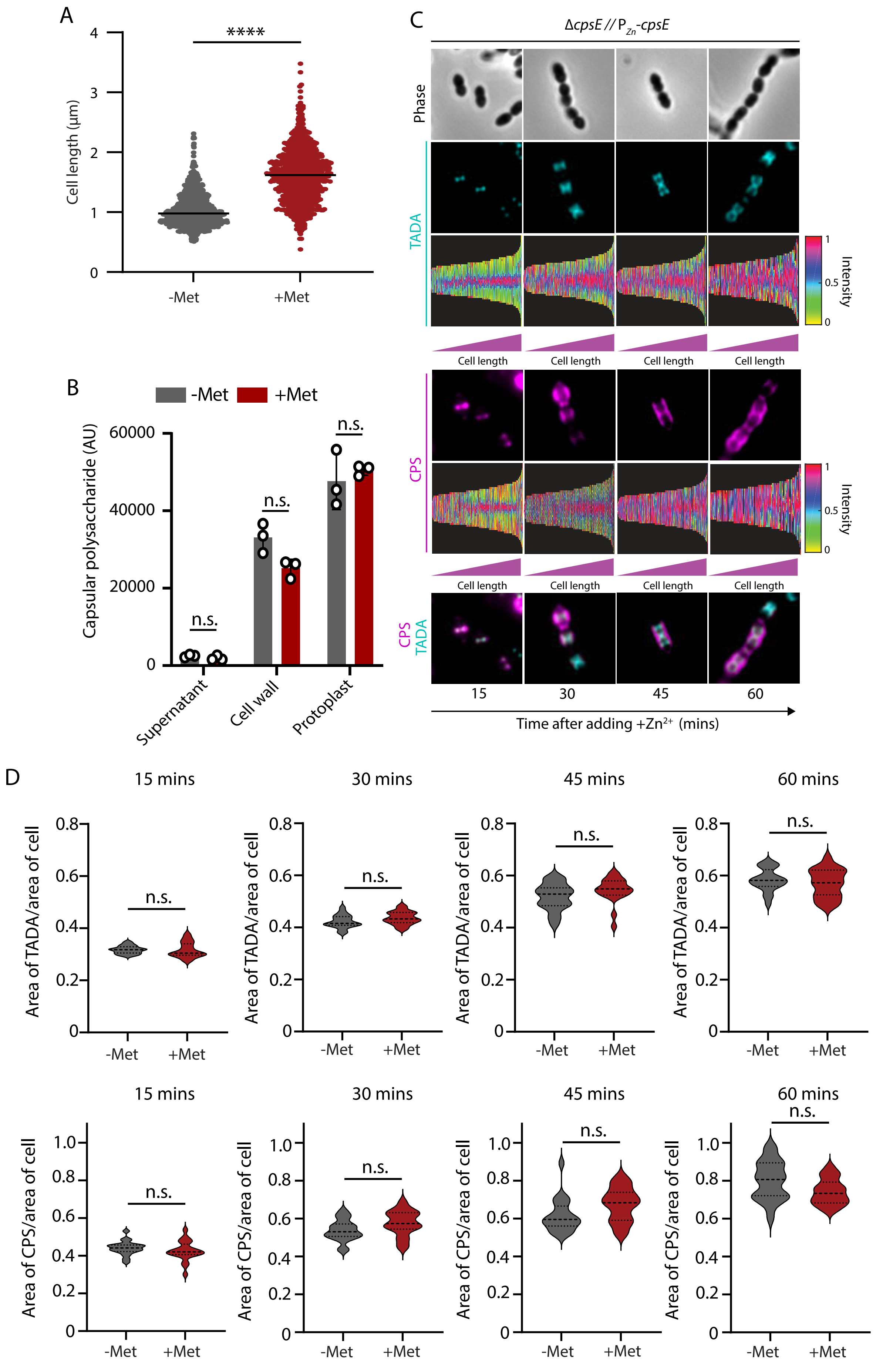

A
Cell length (µm)
****
-Met
+Met
B
-Met
+Met
Capsular polysaccharide (AU)
n.s.
Supernatant
Cell wall
Protoplast
C
ΔcpsE // P_Zn-cpsE
Phase
TADA
CPS
Intensity
Cell length
15
30
45
60
Time after adding +Zn²⁺ (mins)
D
15 mins
30 mins
45 mins
60 mins
Area of TADA/area of cell
Area of CPS/area of cell
n.s.
-Met
+Met

84

Supplemental Fig. S6. Methicillin (Met) treatment does not affect capsule synthesis. (A) Strain IU1781 (isogenic parent) was grown and treated with methicillin as described in the legends of Fig. 4. Cells were collected and imaged with phase-contrast microscopy. The dimension of the cells was measured with Oufti and plotted (-Met *n*=1378; +Met *n*=937). P-value was calculated using two-sided Mann-Whitney U test. ****, p<0.0001. (-Met vs +Met p<0.0001) Error bars represent the mean. (B) The CPS amount in each cellular fraction of cells treated with methicillin. A representative immunoblot is shown in Fig. 4C and the experimental procedure is provided in the legends of Fig. 4 and Methods. CPS was quantified from three biological replicates and no significant change was detected. P values were calculated with two-sided t test. ns, not significant. Data represent mean ± SD from *n*=3 biologically independent experiments. (C) For comparison, the pulse-chase experiment described in Fig. 4A was performed in strain NUS0267 [Δ*cpsE* // P_{Zn}-*cpsE*] but without methicillin treatment. Demograph analysis includes data from three biologically independent experiments (*n*=500). Bar, 2μm. (D) Incorporation of new PG and new CPS at different time points (*n*=20). P values were calculated with two-sided t test. ns, not significant. Error bars represent the SD.

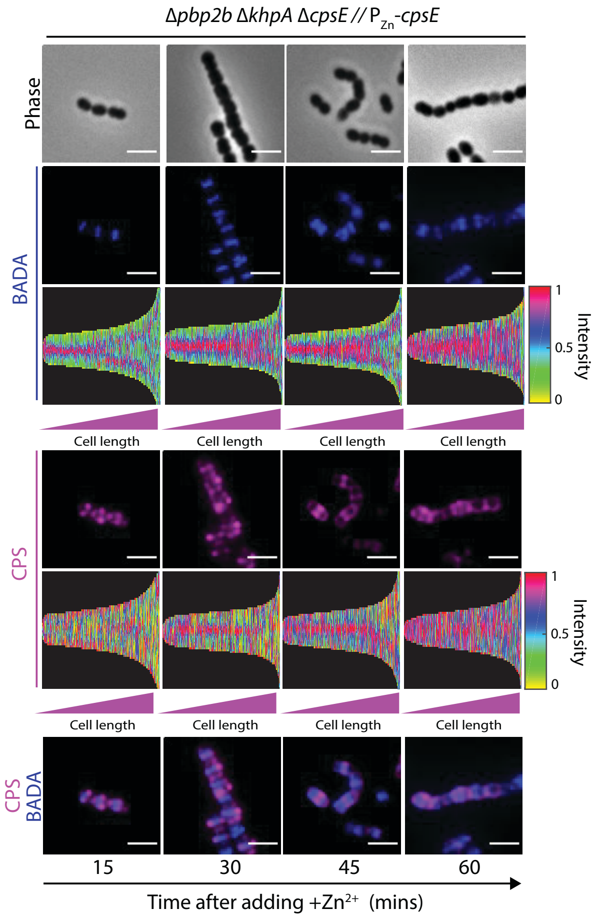

Supplemental Fig. S7. Disruption of Pbp2b does not affect the location of CPS synthesis. The pulse-chase experiment was done with strain NUS1753 [Δ*pbp2B*::P-*kan*-$rpsL^+$ Δ*khpA cpsC-L-sfgfp* Δ*cpsE* // P_{Zn}-*cpsE*] as described in the legends of Fig. 4A. Here, BADA (dark blue) was used instead of TADA and methicillin was not added. Bar, 2μm. Demograph analysis includes data from three biologically independent experiments (*n*=500).

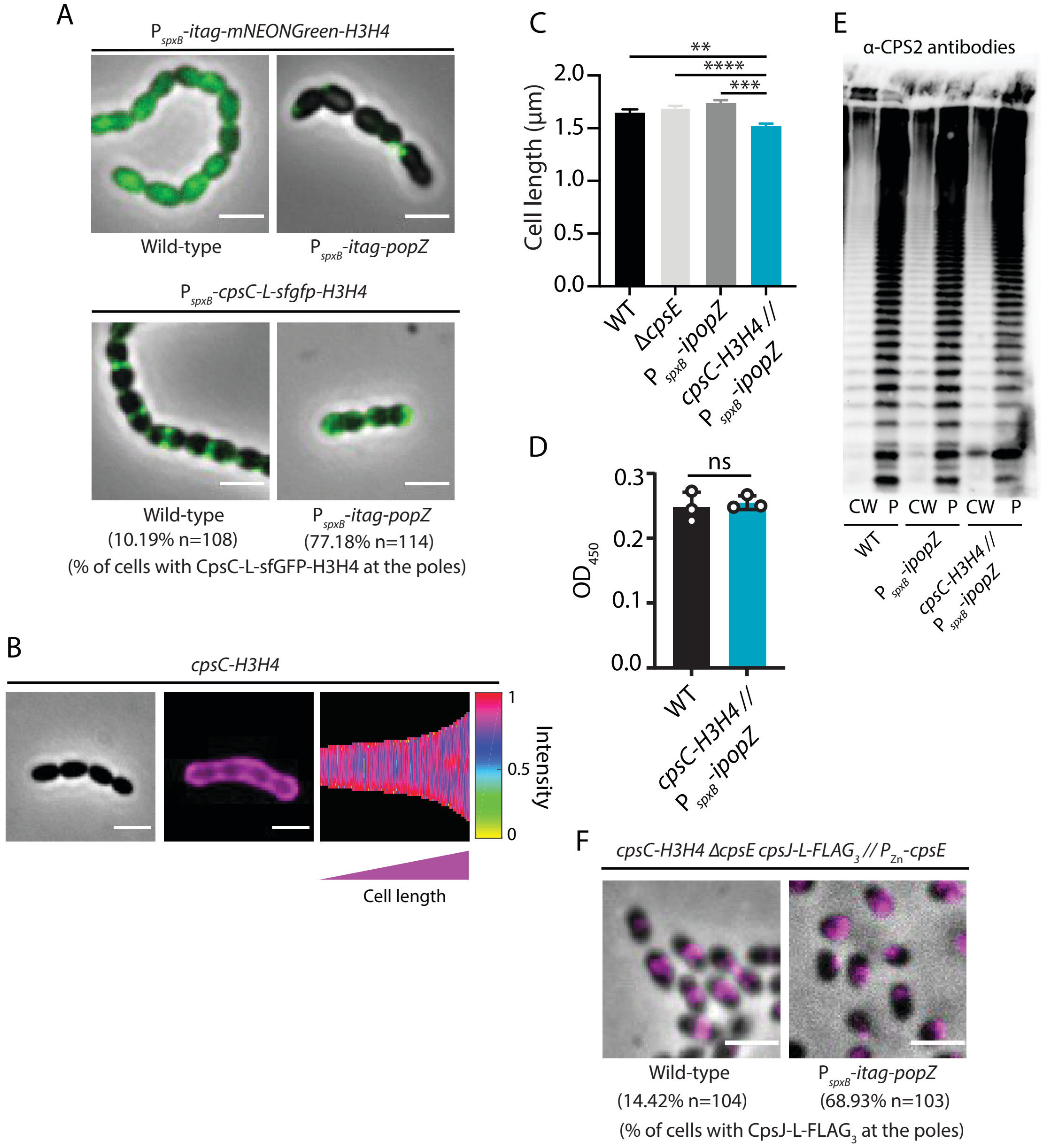

Supplemental Fig. S8. Targeting CpsC to the cell pole disrupts chain formation and modestly affects the cell shape, but it does not change the amount of CPS produced. (A) The POLAR system targets proteins to the cell pole in *S. pneumoniae*. Strains NUS1782 [P$_{spxB}$-*itag-mNEONGreen-H3H4*], NUS1783 [P$_{spxB}$-*cpsC-L-sfgfp-H3H4*], NUS1861 [P$_{spxB}$-*itag-popZ* // P$_{spxB}$-*itag-mNEONGreen-H3H4*], and NUS1862 [P$_{spxB}$-*itag-popZ* // P$_{spxB}$-*cpsC-L-sfgfp-H3H4*] were grown until the cultures reached the mid-log phase. Cells were mounted on a glass slide and visualized with fluorescence microscopy. The percentage of cells with *cpsC-L-sfgfp* at the poles is 10.19% for strain NUS1783 and 77.18% for strain NUS1862. Notably, the number of cells in a chain of strain NUS1862 is different from the wild-type. Bar, 2µm. Experiments were performed independently thrice with similar results. (B) Strain NUS2333 was grown until the cultures reached the mid-log phase. Cells were heat-killed and visualized by

immunostaining using anti-capsule antibodies and fluorescence microscopy. Shown are the representative images (left) and the associated demographs (right). Bar, 2μm. Demograph analysis includes data from two biologically independent experiments (*n*=500). (C) Strains IU1781 (WT), HMS0002 (Δ*cpsE*), NUS2335 [P_{spxB}-*itag-popZ*], and NUS2029 [*cpsC-H3H4* // P_{spxB}-*itag-popZ*] were grown until the culture reached the mid-log phase. Cells were mounted on a glass slide and imaged under phase-contrast microscopy. The dimension of the cells was measured with Oufti and plotted (Wildtype n=236; Δ*cpsE* n=297*; itag-popZ* n=320*; cpsC-H3H4* // *itag-popZ* n=273). P-values were calculated using two-sided Mann-Whitney U-test. **, p<0.01; ***, p<0.005; ****, p<0.001. (Wildtype vs *cpsC-H3H4* // *itag-popZ* p=0.0057 *;* Δ*cpsE* vs *cpsC-H3H4* // *itag-popZ* p=0.0002 *; itag-popZ* vs *cpsC-H3H4* // *itag-popZ* p<0.0001. Error bars represent the mean. (D) Strains IU1781 (isogenic parent; WT) and NUS2029 were cultured until they reached the mid-log phase. Cells were harvested and immobilized on an ELISA plate. CPS was quantified with anti-serotype 2 capsule antisera. Following detection by HRP-linked secondary antibodies and a colorimetric substrate, the signal (OD_{450}) was measured using a plate reader. P values were calculated with two-sided t-test. ns, not significant. Data represent mean ± SD from *n*=3 biologically independent experiments. (E) Alternatively, the collected cells were fractionated into the cell wall (CW) and protoplast (P) fractions. The CPS in each fraction was resolved by SDS-PAGE and detected by immunoblotting as described in Methods. As a negative control, samples from the isogenic parent strain NUS2335 [P_{spxB}-*itag-popZ*] were included. Experiments were performed independently thrice with similar results. (F) Targeting CpsC to the poles changes the localization of CpsJ-L-$FLAG_3$. Strain NUS3893 [*rpsL1* Δ*cpsE cpsC-H3H4 cpsJ-L-FLAG*$_3$ // Δ*bgaA*::P_{Zn}-*cpsE*] and NUS4000 [*rpsL1* Δ*cpsE cpsC-H3H4 cpsJ-L-FLAG*$_3$ // Δ*bgaA*::P_{Zn}-*cpsE* // CEP::P_{spxB}-*itag-popZ*-P-*spec*] was grown until OD_{600} is between 0.2 and 0.4. Cells were collected, immunostained, and visualized under fluorescence microscopy. The percentage of cells with *cpsJ-L-FLAG*$_3$ at the poles is 14.42% (*n*=104) for strain NUS3893 and 68.93% (*n*=103) for strain NUS4000. Experiments were performed independently twice with similar results.

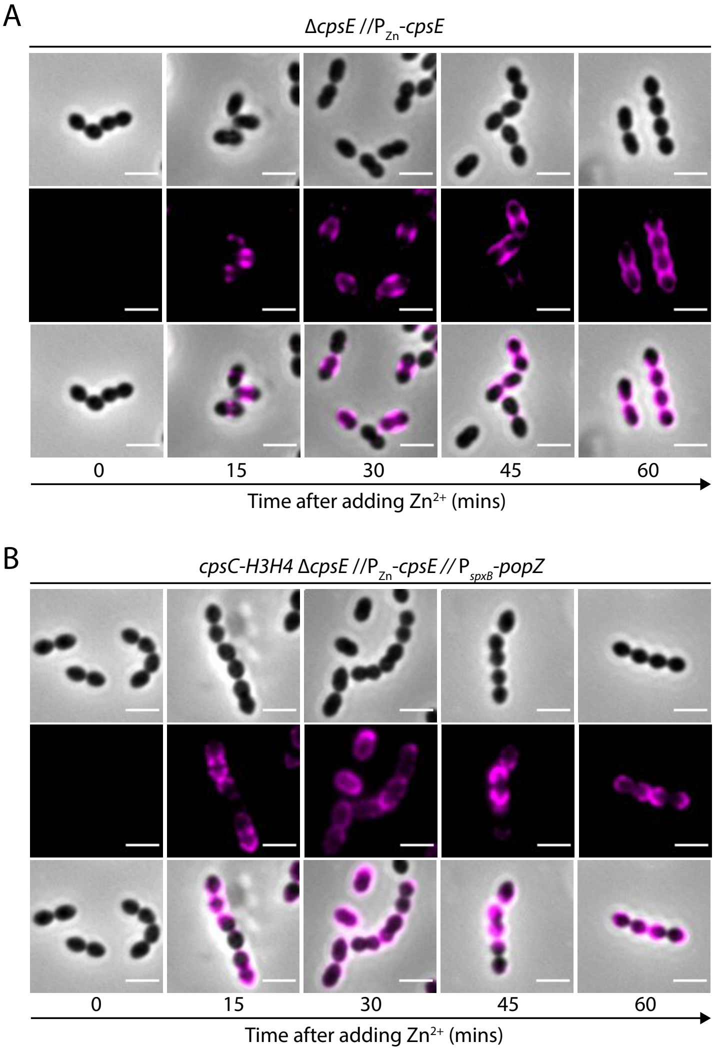

Supplemental Fig. S9. Extra time points of the pulse-chase experiment shown in Fig. 5D. (A) Strain NUS0267 [Δ*cpsE* // P$_{Zn}$-*cpsE*] and (B) NUS3486 [*cpsC-H3H4* Δ*cpsE* // P$_{spxB}$-*itag-popZ* // P$_{Zn}$-*cpsE*] were grown and processed as described in the legends of Fig. 5. The nascent CPS was visualized by immunofluorescence microscopy after the cells were heat-killed at the indicated time points. Bar, 2µm. Experiments were performed independently twice with similar results.

Supplemental Table 1. Strains used in this study.

Strain number	Genotype (descriptions)[a, b]	Derivation	Selectable markers[c]	Source
IU1690	serotype 2 strain D39 (D39W)	-	-	(1, 2)
IU1781	*rpsL1*	*rpsL1* x IU1690	Str^R	(3)
HMS0001	*rpsL1* Δ*cpsE*<>P-*kan-rpsL*$^+$	Δ*cpsE*<>P-*kan-rpsL*$^+$ x IU1781	Kan^R Str^S	(4)
HMS0002	*rpsL1* Δ*cpsE*	Δ*cpsE* x HMS0001	Str^R	(4)
NUS0122	*rpsL1* Δ*cpsE* Δ*bgaA*::P-*sacB-kan-rpsL*$^+$	Δ*bgaA*::P-*sacB-kan-rpsL*$^+$ x IU1781	Suc^S Kan^R Str^S	(4)
NUS0267	*rpsL1* Δ*cpsE* // Δ*bgaA*::P$_{Zn}$-*cpsE*	Δ*bgaA*::P$_{Zn}$-*cpsE* x NUS0122	Str^R	(4)
NUS0426	*rpsL1* Δ*cpsC*::P-*sacB-kan-rpsL*$^+$ Δ*cpsE* // Δ*bgaA*::P$_{Zn}$-*cpsE*	Δ*cpsC*::P-*sacB-kan-rpsL*$^+$ x NUS0267	Suc^S Kan^R Str^S	(4)
NUS1174	*rpsL1 cpsC*^{R121E} Δ*cpsE* // Δ*bgaA*::P$_{Zn}$-*cpsE*	*cpsC*^{R121E} x NUS0426	Str^R	(4)
NUS0882	*rpsL1 cpsC-L-sfgfp* Δ*cpsE* // Δ*bgaA*::P$_{Zn}$-*cpsE*	*cpsC-L-sfgfp* x NUS0426	Str^R	This study
NUS1613	*rpsL1* Δ*cpsE cpsC-L-sfgfp* Δ*bgaA*::P-*sacB-kan-rpsL*$^+$	Δ*bgaA*::P-*sacB-kan-rpsL*$^+$ x NUS0882	Suc^S Kan^R Str^S	This study
NUS1625	*rpsL1 cpsC-L-sfgfp* Δ*cpsE* // Δ*bgaA*::P$_{Zn}$-*ftsZ*	Δ*bgaA*::P$_{Zn}$-*ftsZ* x NUS1613	Str^R	This study
NUS1659	*rpsL1* Δ*ftsZ*::P-*kan-rpsL*$^+$ *cpsC-L-sfgfp* Δ*cpsE* // Δ*bgaA*::P$_{Zn}$-*ftsZ*	Δ*ftsZ*::P-*kan-rpsL*$^+$ x NUS1625	Kan^R Str^S	This study
NUS1706	*rpsL1* Δ*mapZ*::P-*kan-rpsL*$^+$ *cpsC-L-sfgfp* Δ*cpsE* // Δ*bgaA*::P$_{Zn}$-*cpsE*	Δ*mapZ*::P-*kan-rpsL*$^+$ x NUS0882	Kan^R Str^S	This study
NUS3474	*rpsL1* Δ*rocS*::P-*erm cpsC-L-sfgfp* Δ*cpsE* // Δ*bgaA*::P$_{Zn}$-*cpsE*	Δ*rocS*::P-*erm* x NUS0882	Str^R Erm^R	This study
NUS3476	*rpsL1* Δ*ccrZ*::P-*erm cpsC-L-sfgfp* Δ*cpsE* // Δ*bgaA*::P$_{Zn}$-*cpsE*	Δ*ccrZ*::P-*erm* x NUS0882	Str^R Erm^R	This study
NUS3478	*rpsL1* Δ*pbp1A*::P-*erm cpsC-L-sfgfp* Δ*cpsE* // Δ*bgaA*::P$_{Zn}$-*cpsE*	Δ*pbp1A*::P-*erm* x NUS0882	Str^R Erm^R	This study
NUS3487	*rpsL1* Δ*pbp2A*::P-*erm cpsC-L-sfgfp* Δ*cpsE* // Δ*bgaA*::P$_{Zn}$-*cpsE*	Δ*pbp2A*::P-*erm* x NUS0882	Str^R Erm^R	This study
NUS3505	*rpsL1 pbp1B*::P-*erm cpsC-L-sfgfp* Δ*cpsE* // Δ*bgaA*::P$_{Zn}$-*cpsE*	*pbp1B*::P-*erm* x NUS0882	Str^R Erm^R	This study
NUS1858	*rpsL1* Δ*cpsE cpsC-L-sfgfp* // Δ*bgaA*::P$_{Zn}$-*ezrA*	Δ*bgaA*::P$_{Zn}$-*ezrA* x NUS1613	Str^R	This study
NUS1869	*rpsL1 cpsC-L-sfgfp* Δ*cpsE* Δ*ezrA*::P-*kan-rpsL*$^+$ // Δ*bgaA*::P$_{Zn}$-*ezrA*	Δ*ezrA*::P-*kan-rpsL*$^+$ x NUS1858	Str^R	This study
NUS1674	*rpsL1 cpsC-L-sfgfp* Δ*cpsE* // Δ*bgaA*::P$_{Zn}$-*ftsX*	Δ*bgaA*::P$_{Zn}$-*ftsX* x NUS1613	Str^R	This study
NUS1675	*rpsL1 cpsC-L-sfgfp* Δ*cpsE* // Δ*bgaA*::P$_{Zn}$-*ftsQ*	Δ*bgaA*::P$_{Zn}$-*ftsQ* x NUS1613	Str^R	This study
NUS1676	*rpsL1 cpsC-L-sfgfp* Δ*cpsE* // Δ*bgaA*::P$_{Zn}$-*pbp2X*	Δ*bgaA*::P$_{Zn}$-*pbp2X* x NUS1613	Str^R	This study
NUS1677	*rpsL1 cpsC-L-sfgfp* Δ*cpsE* // Δ*bgaA*::P$_{Zn}$-*pbp2B*	Δ*bgaA*::P$_{Zn}$-*pbp2B* x NUS1613	Str^R	This study
NUS1810	*rpsL1 cpsC-L-sfgfp* Δ*cpsE* Δ*bgaA*::P$_{Zn}$-*ftsW*	Δ*bgaA*::P$_{Zn}$-*ftsW* x NUS1613	Str^R	This study
NUS1749	*rpsL1 cpsC-L-sfgfp* Δ*cpsE* Δ*ftsX*::P-*kan-rpsL*$^+$ // Δ*bgaA*::P$_{Zn}$-*ftsX*	Δ*ftsX*::P-*kan-rpsL*$^+$ x NUS1674	Kan^R Str^S	This study

NUS1750	*rpsL1 cpsC-L-sfgfp* Δ*cpsE* Δ*ftsQ*::P-*kan-rpsL*$^+$ // Δ*bgaA*::P$_{Zn}$-*ftsQ*	Δ*ftsQ*::P-*kan-rpsL*$^+$ x NUS1675	Kan^R Str^S	This study
NUS1751	*rpsL1 cpsC-L-sfgfp* Δ*cpsE* Δ*pbp2X*::P-*kan-rpsL*$^+$ // Δ*bgaA*::P$_{Zn}$-*pbp2X*	Δ*pbp2X*::P-*kan-rpsL*$^+$ x NUS1676	Kan^R Str^S	This study
NUS1775	*rpsL1 cpsC-L-sfgfp* Δ*cpsE* Δ*pbp2B*::P-*kan-rpsL*$^+$ // Δ*bgaA*::P$_{Zn}$-*pbp2B*	Δ*pbp2B*::P-*kan-rpsL*$^+$ x NUS1677	Kan^R Str^S	This study
NUS1826	*rpsL1 cpsC-L-sfgfp* Δ*cpsE* Δ*ftsW*::P-*kan-rpsL*$^+$ // Δ*bgaA*::P$_{Zn}$-*ftsW*	Δ*ftsW*::P-*kan-rpsL*$^+$ x NUS1810	Kan^R Str^S	This study
NUS1704	*rpsL1 cpsC-L-sfgfp* Δ*cpsE* Δ*khpA*::P-*sacB-kan-rpsL*$^+$ // Δ*bgaA*::P$_{Zn}$-*cpsE*	Δ*khpA*::P-*sacB-kan-rpsL*+ x NUS0882	Suc^S Kan^R Str^S	This study
NUS1717	*rpsL1 cpsC-L-sfgfp* Δ*cpsE* Δ*khpA* // Δ*bgaA*::P$_{Zn}$-*cpsE*	Δ*khpA* x NUS1704	Str^R	This study
NUS1740	*rpsL1* Δ*gpsB*::P-*kan-rpsL*$^+$ Δ*khpA cpsC-L-sfgfp* Δ*cpsE* // Δ*bgaA*::P$_{Zn}$-*cpsE*	Δ*gpsB*::P-*kan-rpsL*$^+$ x NUS1717	Kan^R Str^S	This study
NUS1748	*rpsL1* Δ*mreC*::P-*kan-rpsL*$^+$ Δ*khpA cpsC-L-sfgfp* Δ*cpsE* // Δ*bgaA*::P$_{Zn}$-*cpsE*	Δ*mreC*::P-*kan-rpsL*$^+$ x NUS1717	Kan^R Str^S	This study
NUS1753	*rpsL1* Δ*pbp2B*::P-*kan-rpsL*$^+$ Δ*khpA cpsC-L-sfgfp* Δ*cpsE* // Δ*bgaA*::P$_{Zn}$-*cpsE*	Δ*pbp2B*::P-*kan-rpsL*$^+$ x NUS1717	Kan^R Str^S	This study
NUS1806	*rpsL1* Δ*rodA*::P-*kan-rpsL*$^+$ Δ*khpA cpsC-L-sfgfp* Δ*cpsE* // Δ*bgaA*::P$_{Zn}$-*cpsE*	Δ*rodA*::P-*kan-rpsL*$^+$ x NUS1717	Kan^R Str^S	This study
NUS0422	*rpsL1* Δ*cpsCDE*::P-*sacB-kan-rpsL*$^+$	Δ*cpsCDE*::P-*sacB-kan-rpsL*$^+$ x IU1781	Suc^S Kan^R Str^S	This study
NUS1571	*rpsL1 cpsC-L-FLAG*$_3$	*cpsC-L-FLAG*$_3$ (*cpsDE*$^+$) x NUS0422	Str^R	This study
NUS1611	*rpsL1 cpsC-L-FLAG*$_3$ // Δ*bgaA*::P-*sacB-kan-rpsL*$^+$	Δ*bgaA*::P-*sacB-kan-rpsL*$^+$ x NUS1571	Suc^S Kan^R Str^S	This study
NUS1624	*rpsL1 cpsC-L-FLAG*$_3$ // Δ*bgaA*::P$_{Zn}$-*ftsZ*	Δ*bgaA*::P$_{Zn}$-*ftsZ* x NUS1611	Str^R	This study
NUS1640	*rpsL1 cpsC-L-FLAG*$_3$ Δ*ftsZ*::P-*kan-rpsL*$^+$ // Δ*bgaA*::P$_{Zn}$-*ftsZ*	Δ*ftsZ*::P-*kan-rpsL*$^+$ x NUS1624	Kan^R Str^S	This study
NUS1680	*rpsL1 cpsC-L-FLAG*$_3$ // Δ*bgaA*::P$_{Zn}$-*pbp2X*	Δ*bgaA*::P$_{Zn}$-*pbp2X* x NUS1611	Str^R	This study
NUS1679	*rpsL1 cpsC-L-FLAG*$_3$ // Δ*bgaA*::P$_{Zn}$-*ftsQ*	*bgaA*::P$_{Zn}$-*ftsQ* x NUS1611	Str^R	This study
NUS1860	*rpsL1 cpsC-L-FLAG*$_3$ // Δ*bgaA*::P$_{Zn}$-*ftsW*	*bgaA*::P$_{Zn}$-*ftsW* x NUS1611	Str^R	This study
NUS1915	*rpsL1 cpsC-L-FLAG*$_3$ *ftsZ*$^+$-P-*kan-rpsL*$^+$ // *bgaA*::P$_{Zn}$-*pbp2x*	*ftsZ*$^+$-P-*kan-rpsL*$^+$ x NUS1680	Kan^R Str^S	This study
NUS1914	*rpsL1 cpsC-L-FLAG*$_3$ *ftsZ*$^+$-P-*kan-rpsL*$^+$ // *bgaA*::P$_{Zn}$-*ftsQ*	*ftsZ*$^+$-P-*kan-rpsL*$^+$ x NUS1679	Kan^R Str^S	This study
NUS1918	*rpsL1 cpsC-L-FLAG*$_3$ *ftsZ*$^+$-P-*kan-rpsL*$^+$ // *bgaA*::P$_{Zn}$-*ftsW*	*ftsZ*$^+$-P-*kan-rpsL*$^+$ x NUS1860	Kan^R Str^S	This study
NUS2034	*rpsL1 cpsC-L-FLAG*$_3$ *ftsZ-L-sfgfp* // *bgaA*::P$_{Zn}$-*pbp2x*	*ftsZ-L-sfgfp* x NUS1915	Str^R	This study
NUS2033	*rpsL1 cpsC-L-FLAG*$_3$ *ftsZ-L-sfgfp* // *bgaA*::P$_{Zn}$-*ftsQ*	*ftsZ-L-sfgfp* x NUS1914	Str^R	This study
NUS2037	*rpsL1 cpsC-L-FLAG*$_3$ *ftsZ-L-sfgfp* // *bgaA*::P$_{Zn}$-*ftsW*	*ftsZ-L-sfgfp* x NUS1918	Str^R	This study

NUS2185	*rpsL1* Δ*pbp2X*::P-*kan-rpsL*$^+$ *cpsC-L-FLAG*$_3$ *ftsZ-L-sfgfp* // *bgaA*::P$_{Zn}$-*pbp2x*	Δ*pbp2X*::P-*kan-rpsL*$^+$ x NUS2034	Kan^R Str^S	This study
NUS2068	*rpsL1* Δ*ftsQ*::P-*kan-rpsL*$^+$ *cpsC-L-FLAG*$_3$ *ftsZ-L-sfgfp* // *bgaA*::P$_{Zn}$-*ftsQ*	Δ*ftsQ*::P-*kan-rpsL*$^+$ x NUS2033	Kan^R Str^S	This study
NUS2184	*rpsL1* Δ*ftsW*::P-*kan-rpsL*$^+$ *cpsC-L-FLAG*$_3$ *ftsZ-L-sfgfp* // *bgaA*::P$_{Zn}$-*ftsW*	Δ*ftsW*::P-*kan-rpsL*$^+$ x NUS2037	Kan^R Str^S	This study
NUS2186	*rpsL1* Δ*khpA*::P-*kan-rpsL*$^+$	Δ*khpA*::P-*kan-rpsL*$^+$ x IU1781	Kan^R Str^S	This study
NUS3454	*rpsL1* Δ*pbp2B*::P-*erm* Δ*khpA*::P-*kan-rpsL*$^+$	Δ*pbp2B*::P-*erm* x NUS2186	Erm^R Kan^R Str^S	This study
NUS1090	*rpsL1 cpsC-L-FLAG*$_3$ Δ*cpsE* // Δ*bgaA*::P$_{Zn}$-*cpsE*	*cpsC-L-FLAG*$_3$ x NUS0426	Str^R	This study
NUS2235	*rpsL1* Δ*cpsA*::P-*sacB-kan-rpsL*$^+$ *cpsC-L-FLAG*$_3$ Δ*cpsE* // Δ*bgaA*::P$_{Zn}$-*cpsE*	Δ*cpsA*::P-*sacB-kan-rpsL*$^+$ x NUS1090	Suc^S Kan^R Str^S	This study
NUS2236	*rpsL1* Δ*cpsB*::P-*sacB-kan-rpsL*$^+$ *cpsC-L-FLAG*$_3$ Δ*cpsE* // Δ*bgaA*::P$_{Zn}$-*cpsE*	Δ*cpsB*::P-*sacB-kan-rpsL*$^+$ x NUS1090	Suc^S Kan^R Str^S	This study
NUS2307	*rpsL1* Δ*cpsD*::P-*sacB-kan-rpsL*$^+$ *cpsC-L-FLAG*$_3$ Δ*cpsE* // Δ*bgaA*::P$_{Zn}$-*cpsE*	Δ*cpsD*::P-*sacB-kan-rpsL*$^+$ x NUS1090	Suc^S Kan^R Str^S	This study
NUS2238	*rpsL1* Δ*cpsG*::P-*sacB-kan-rpsL*$^+$ *cpsC-L-FLAG*$_3$ Δ*cpsE* // Δ*bgaA*::P$_{Zn}$-*cpsE*	Δ*cpsG*::P-*sacB-kan-rpsL*$^+$ x NUS1090	Suc^S Kan^R Str^S	This study
NUS2239	*rpsL1* Δ*cpsI*::P-*sacB-kan-rpsL*$^+$ *cpsC-L-FLAG*$_3$ Δ*cpsE* // Δ*bgaA*::P$_{Zn}$-*cpsE*	Δ*cpsI*::P-*sacB-kan-rpsL*$^+$ x NUS1090	Suc^S Kan^R Str^S	This study
NUS2240	*rpsL1* Δ*cpsK*::P-*sacB-kan-rpsL*$^+$ *cpsC-L-FLAG*$_3$ Δ*cpsE* // Δ*bgaA*::P$_{Zn}$-*cpsE*	Δ*cpsK*::P-*sacB-kan-rpsL*$^+$ x NUS1090	Suc^S Kan^R Str^S	This study
NUS2647	*rpsL1* Δ*cpsJ*::P-*sacB-kan-rpsL*$^+$ *cpsC-L-FLAG*$_3$ Δ*cpsE* // Δ*bgaA*::P$_{Zn}$-*cpsE*	Δ*cpsJ*::P-*sacB-kan-rpsL*$^+$ x NUS1090	Suc^S Kan^R Str^S	This study
NUS2648	*rpsL1* Δ*cpsH*::P-*sacB-kan-rpsL*$^+$ *cpsC-L-FLAG*$_3$ Δ*cpsE* // Δ*bgaA*::P$_{Zn}$-*cpsE*	Δ*cpsH*::P-*sacB-kan-rpsL*$^+$ x NUS1090	Suc^S Kan^R Str^S	This study
NUS2649	*rpsL1* Δ*cpsP*::P-*sacB-kan-rpsL*$^+$ *cpsC-L-FLAG*$_3$ Δ*cpsE* // Δ*bgaA*::P$_{Zn}$-*cpsE*	Δ*cpsP*::P-*sacB-kan-rpsL*$^+$ x NUS1090	Suc^S Kan^R Str^S	This study
NUS2650	*rpsL1* Δ*cpsL*::P-*sacB-kan-rpsL*$^+$ *cpsC-L-FLAG*$_3$ Δ*cpsE* // Δ*bgaA*::P$_{Zn}$-*cpsE*	Δ*cpsL*::P-*sacB-kan-rpsL*$^+$ x NUS1090	Suc^S Kan^R Str^S	This study
NUS2652	*rpsL1* Δ*cpsT*::P-*sacB-kan-rpsL*$^+$ *cpsC-L-FLAG*$_3$ Δ*cpsE* // Δ*bgaA*::P$_{Zn}$-*cpsE*	Δ*cpsT*::P-*sacB-kan-rpsL*$^+$ x NUS1090	Suc^S Kan^R Str^S	This study
NUS0679	*rpsL1* Δ*cpsC* Δ*cpsE* // Δ*bgaA*::P$_{Zn}$-*cpsE*	Δ*cpsC* x NUS0426	Str^R	
NUS2296	*rpsL1* Δ*cpsA*::P-*sacB-kan-rpsL*$^+$ Δ*cpsC* Δ*cpsE* // Δ*bgaA*::P$_{Zn}$-*cpsE*	Δ*cpsA*::P-*sacB-kan-rpsL*$^+$ x NUS0679	Suc^S Kan^R Str^S	This study
NUS3100	*rpsL1 cpsA-L-FLAG*$_3$ Δ*cpsC* Δ*cpsE* // Δ*bgaA*::P$_{Zn}$-*cpsE*	*cpsA-L-FLAG*$_3$ x NUS2296	Str^R	This study
NUS0546	*rpsL1* Δ*cpsA*::P-*sacB-kan-rpsL*$^+$ Δ*cpsE* // Δ*bgaA*::P$_{Zn}$-*cpsE*	Δ*cpsA*::P-*sacB-kan-rpsL*$^+$ x NUS0267	Suc^S Kan^R Str^S	This study

NUS0734	*rpsL1 cpsA-L-FLAG$_3$ ΔcpsE* // *ΔbgaA*::P$_{Zn}$-*cpsE*	*cpsA-L-FLAG$_3$* x NUS0546	Str^R	This study
NUS3325	*rpsL1 cpsA-L-FLAG$_3$ ΔcpsB*::P-*erm ΔcpsE* // *ΔbgaA*::P$_{Zn}$-*cpsE*	*ΔcpsB*::P-*erm* x NUS0734	Str^R Erm^R	This study
NUS3279	*rpsL1 cpsA-L-FLAG$_3$ ΔcpsD*::P-*sacB-kan-rpsL$^+$ ΔcpsE* // *ΔbgaA*::P$_{Zn}$-*cpsE*	*ΔcpsD*::P-*sacB-kan-rpsL$^+$* x NUS0734	Suc^S Kan^R Str^S	This study
NUS3375	*rpsL1 cpsA-L-FLAG$_3$ ΔcpsE ΔcpsG*::P-*erm* // *ΔbgaA*::P$_{Zn}$-*cpsE*	*ΔcpsG*::P-*erm* x NUS0734	Str^R Erm^R	This study
NUS3326	*rpsL1 cpsA-L-FLAG$_3$ ΔcpsE ΔcpsI*::P-*erm* // *ΔbgaA*::P$_{Zn}$-*cpsE*	*ΔcpsI*::P-*erm* x NUS0734	Str^R Erm^R	This study
NUS3282	*rpsL1 cpsA-L-FLAG$_3$ ΔcpsE ΔcpsK*::P-*sacB-kan-rpsL$^+$* // *ΔbgaA*::P$_{Zn}$-*cpsE*	*ΔcpsK*::P-*sacB-kan-rpsL$^+$* x NUS0734	Suc^S Kan^R Str^S	This study
NUS3408	*rpsL1 cpsA-L-FLAG$_3$ ΔcpsE ΔcpsJ*:: P-*erm* // *ΔbgaA*::P$_{Zn}$-*cpsE*	*ΔcpsJ*:: P-*erm* x NUS0734	Str^R Erm^R	This study
NUS3283	*rpsL1 cpsA-L-FLAG$_3$ ΔcpsE ΔcpsH*::P-*sacB-kan-rpsL$^+$* // *ΔbgaA*::P$_{Zn}$-*cpsE*	*ΔcpsH*::P-*sacB-kan-rpsL$^+$* x NUS0734	Suc^S Kan^R Str^S	This study
NUS3284	*rpsL1 cpsA-L-FLAG$_3$ ΔcpsE ΔcpsP*::P-*sacB-kan-rpsL$^+$* // *ΔbgaA*::P$_{Zn}$-*cpsE*	*ΔcpsP*::P-*sacB-kan-rpsL$^+$* x NUS0734	Suc^S Kan^R Str^S	This study
NUS3285	*rpsL1 cpsA-L-FLAG$_3$ ΔcpsE ΔcpsL*::P-*sacB-kan-rpsL$^+$* // *ΔbgaA*::P$_{Zn}$-*cpsE*	*ΔcpsL*::P-*sacB-kan-rpsL$^+$* x NUS0734	Suc^S Kan^R Str^S	This study
NUS3327	*rpsL1 cpsA-L-FLAG$_3$ ΔcpsE ΔcpsT*::P-*sacB-kan-rpsL$^+$* // *ΔbgaA*::P$_{Zn}$-*cpsE*	*ΔcpsT*::P-*sacB-kan-rpsL$^+$* x NUS0734	Suc^S Kan^R Str^S	This study
NUS0713	*rpsL1 ΔcpsE ΔcpsJ*::P-*sacB-kan-rpsL$^+$* // *ΔbgaA*::P$_{Zn}$-*cpsE*	*ΔcpsJ*::P-*sacB-kan-rpsL$^+$* x NUS0267	Suc^S Kan^R Str^S	This study
NUS3063	*rpsL1 ΔcpsE cpsJ-L-FLAG$_3$* // *ΔbgaA*::P$_{Zn}$-*cpsE*	*cpsJ-L-FLAG$_3$* x NUS0713	Str^R	This study
NUS2302	*rpsL1 ΔcpsJ*::P-*sacB-kan-rpsL$^+$ ΔcpsC ΔcpsE* // *ΔbgaA*::P$_{Zn}$-*cpsE*	*ΔcpsJ*::P-*sacB-kan-rpsL$^+$* X NUS0679	Suc^S Kan^R Str^S	This study
NUS3065	*rpsL1 cpsJ-L-FLAG$_3$ ΔcpsC ΔcpsE* // *ΔbgaA*::P$_{Zn}$-*cpsE*	*cpsJ-L-FLAG$_3$* x NUS2302	Str^R	This study
NUS3286	*rpsL1 ΔcpsB*::P-*sacB-kan-rpsL$^+$ cpsJ-L-FLAG$_3$ ΔcpsE* // *ΔbgaA*::P$_{Zn}$-*cpsE*	*ΔcpsB*::P-*sacB-kan-rpsL$^+$* x NUS3063	Suc^S Kan^R Str^S	This study
NUS3287	*rpsL1 ΔcpsD*::P-*sacB-kan-rpsL$^+$ cpsJ-L-FLAG$_3$ ΔcpsE* // *ΔbgaA*::P$_{Zn}$-*cpsE*	*ΔcpsD*::P-*sacB-kan-rpsL$^+$* x NUS3063	Suc^S Kan^R Str^S	This study
NUS3384	*rpsL1 ΔcpsG*::P-*erm cpsJ-L-FLAG$_3$ ΔcpsE* // *ΔbgaA*::P$_{Zn}$-*cpsE*	*ΔcpsG*::P-*erm* x NUS3063	Str^R Erm^R	This study
NUS3378	*rpsL1 ΔcpsI*:: P-*erm cpsJ-L-FLAG$_3$ ΔcpsE* // *ΔbgaA*::P$_{Zn}$-*cpsE*	*ΔcpsI*::P-*erm* x NUS3063	Str^R Erm^R	This study
NUS3290	*rpsL1 ΔcpsK*::P-*sacB-kan-rpsL$^+$ cpsJ-L-FLAG$_3$ ΔcpsE* // *ΔbgaA*::P$_{Zn}$-*cpsE*	*ΔcpsK*::P-*sacB-kan-rpsL$^+$* x NUS3063	Suc^S Kan^R Str^S	This study
NUS3291	*rpsL1 ΔcpsH*::P-*sacB-kan-rpsL$^+$ cpsJ-L-FLAG$_3$ ΔcpsE* // *ΔbgaA*::P$_{Zn}$-*cpsE*	*ΔcpsH*::P-*sacB-kan-rpsL$^+$* x NUS3063	Suc^S Kan^R Str^S	This study

NUS3292	*rpsL1* Δ*cpsP*::P-*sacB-kan-rpsL*$^+$ *cpsJ-L-FLAG$_3$* Δ*cpsE* // Δ*bgaA*::P$_{Zn}$-*cpsE*	Δ*cpsP*::P-*sacB-kan-rpsL*$^+$ x NUS3063	Suc^S Kan^R Str^S	This study
NUS3293	*rpsL1* Δ*cpsL*::P-*sacB-kan-rpsL*$^+$ *cpsJ-L-FLAG$_3$* Δ*cpsE* // Δ*bgaA*::P$_{Zn}$-*cpsE*	Δ*cpsL*::P-*sacB-kan-rpsL*$^+$ x NUS3063	Suc^S Kan^R Str^S	This study
NUS3377	*rpsL1* Δ*cpsT*::P-*sacB-kan-rpsL*$^+$ *cpsJ-L-FLAG$_3$* Δ*cpsE* // Δ*bgaA*::P$_{Zn}$-*cpsE*	Δ*cpsT*::P-*sacB-kan-rpsL*$^+$ x NUS3063	Suc^S Kan^R Str^S	This study
NUS1137	*rpsL1* Δ*cpsE* Δ*cpsH*::P-*sacB-kan-rpsL*$^+$ // Δ*bgaA*::P$_{Zn}$-*cpsE*	Δ*cpsH*::P-*sacB-kan-rpsL*$^+$ x NUS0267	Suc^S Kan^R Str^S	This study
NUS1327	*rpsL1* Δ*cpsC* Δ*cpsE* Δ*cpsH*::P-*sacB-kan-rpsL*$^+$ // Δ*bgaA*::P$_{Zn}$-*cpsE*	Δ*cpsH*::P-*sacB-kan-rpsL*$^+$ x NUS0679	Suc^S Kan^R Str^S	This study
NUS3064	*rpsL1* *cpsH-L-FLAG$_3$* Δ*cpsE* // Δ*bgaA*::P$_{Zn}$-*cpsE*	*cpsH-L-FLAG$_3$* x NUS1137	Str^R	This study
NUS3103	*rpsL1* *cpsH-L-FLAG$_3$* Δ*cpsC* Δ*cpsE* // Δ*bgaA*::P$_{Zn}$-*cpsE*	*cpsH-L-FLAG$_3$* x NUS1327	Str^R	This study
NUS3385	*rpsL1* Δ*cpsA*:: P-*sacB-kan-rpsL*$^+$ *cpsH-L-FLAG$_3$* Δ*cpsE* // Δ*bgaA*::P$_{Zn}$-*cpsE*	Δ*cpsA*::P-*sacB-kan-rpsL*$^+$ x NUS3064	Suc^S Kan^R Str^S	This study
NUS3294	*rpsL1* Δ*cpsB*::P-*erm* *cpsH-L-FLAG$_3$* Δ*cpsE* // Δ*bgaA*::P$_{Zn}$-*cpsE*	Δ*cpsB*::P-*erm* x NUS3064	Str^R Erm^R	This study
NUS3295	*rpsL1* Δ*cpsD*::P-*sacB-kan-rpsL*$^+$ *cpsH-L-FLAG$_3$* Δ*cpsE* // Δ*bgaA*::P$_{Zn}$-*cpsE*	Δ*cpsD*::P-*sacB-kan-rpsL*$^+$ x NUS3064	Suc^S Kan^R Str^S	This study
NUS3388	*rpsL1* Δ *cpsG*::P-*erm* *cpsH-L-FLAG$_3$* Δ*cpsE* // Δ*bgaA*::P$_{Zn}$-*cpsE*	Δ*cpsG*::P-*erm* x NUS3064	Str^R Erm^R	This study
NUS3387	*rpsL1* Δ*cpsI*::P-*erm* *cpsH-L-FLAG$_3$* Δ*cpsE* // Δ*bgaA*::P$_{Zn}$-*cpsE*	Δ*cpsI*::P-*erm* x NUS3064	Str^R Erm^R	This study
NUS3298	*rpsL1* Δ*cpsK*::P-*sacB-kan-rpsL*$^+$ *cpsH-L-FLAG$_3$* Δ*cpsE* // Δ*bgaA*::P$_{Zn}$-*cpsE*	Δ*cpsK*::P-*sacB-kan-rpsL*$^+$ x NUS3064	Suc^S Kan^R Str^S	This study
NUS3409	*rpsL1* Δ*cpsJ*:: P-*erm* *cpsH-L-FLAG$_3$* Δ*cpsE* // Δ*bgaA*::P$_{Zn}$-*cpsE*	Δ*cpsJ*:: P-*erm* x NUS3064	Str^R Erm^R	This study
NUS3299	*rpsL1* Δ*cpsP*::P-*sacB-kan-rpsL*$^+$ *cpsH-L-FLAG$_3$* Δ*cpsE* // Δ*bgaA*::P$_{Zn}$-*cpsE*	Δ*cpsP*::P-*sacB-kan-rpsL*$^+$ x NUS0734	Suc^S Kan^R Str^S	This study
NUS3300	*rpsL1* Δ*cpsL*::P-*sacB-kan-rpsL*$^+$ *cpsH-L-FLAG$_3$* Δ*cpsE* // Δ*bgaA*::P$_{Zn}$-*cpsE*	Δ*cpsL*::P-*sacB-kan-rpsL*$^+$ x NUS0734	Suc^S Kan^R Str^S	This study
NUS3386	*rpsL1* Δ*cpsT*::P-*sacB-kan-rpsL*$^+$ *cpsH-L-FLAG$_3$* Δ*cpsE* // Δ*bgaA*::P$_{Zn}$-*cpsE*	Δ*cpsT*::P-*sacB-kan-rpsL*$^+$ x NUS0734	Suc^S Kan^R Str^S	This study
NUS0092	*rpsL1* Δ*bgaA*::P-*spec-rpsL*$^+$	Δ*bgaA*::P-*spec-rpsL*$^+$ x IU1781	Spec^R	This study
NUS3875	*rpsL1* *cpsJ-L-FLAG$_3$* Δ*cpsC* Δ*cpsE* // Δ*bgaA*::P$_{spxB}$-*cpsC*-P-*spec*	Δ*bgaA*::P$_{spxB}$-*cpsC*-P-*spec* x NUS3065	Str^R Spec^R	This study
NUS3876	*rpsL1* *cpsA-L-FLAG$_3$* Δ*cpsC* Δ*cpsE* // Δ*bgaA*::P$_{spxB}$-*cpsC*-P-*spec*	Δ*bgaA*::P$_{spxB}$-*cpsC*-P-*spec* x NUS3100	Str^R Spec^R	This study
NUS3877	*rpsL1* *cpsH-L-FLAG$_3$* Δ*cpsC* Δ*cpsE* // Δ*bgaA*::P$_{spxB}$-*cpsC*-P-*spec*	Δ*bgaA*::P$_{spxB}$-*cpsC*-P-*spec* x NUS3103	Str^R Spec^R	This study

NUS3878	*rpsL1 ΔcpsT*::P-*sacB-kan-rpsL*$^{+}$ *cpsJ-L-FLAG*$_{3}$ *ΔcpsE* // *ΔbgaA*::P$_{spxB}$-*cpsT*-P-*spec*	*ΔbgaA*::P$_{spxB}$-*cpsT*-P-*spec* x NUS3377	Suc^{S} Kan^{R} Str^{S} Spec^{R}	This study
NUS3879	*rpsL1 ΔcpsI*:: P-*erm cpsJ-L-FLAG*$_{3}$ *ΔcpsE* // *ΔbgaA*::P$_{spxB}$-*cpsI*-P-*spec*	*ΔbgaA*::P$_{spxB}$-*cpsI*-P-*spec* x NUS3378	Str^{R} Erm^{R} Spec^{R}	This study
NUS3894	*rpsL1 ΔcpsG*::P-*erm cpsJ-L-FLAG*$_{3}$ *ΔcpsE* // *ΔbgaA*::P$_{spxB}$-*cpsG*-P-*spec*	*ΔbgaA*::P$_{spxB}$-*cpsG*-P-*spec* x NUS3384	Str^{R} Erm^{R} Spec^{R}	This study
NUS0121	*rpsL1 ΔbgaA*::P-*sacB-kan-rpsL*$^{+}$	*ΔbgaA*::P-*sacB-kan-rpsL*$^{+}$ x IU1781	Suc^{S} Kan^{R} Str^{S}	(4)
NUS1423	*rpsL1 ΔbgaA*::P$_{spxB}$-*itag-mNEONGreen*	*ΔbgaA*::P$_{spxB}$-*itag-mNEONGreen* x IU1781	Str^{R}	This study
NUS1782	*rpsL1 ΔbgaA*::P$_{spxB}$-*itag-mNEONGreen-H3H4*	*ΔbgaA*::P$_{spxB}$-*itag-mNEONGreen-H3H4* x NUS0121	Str^{R}	This study
NUS1836	*rpsL1 CEP*::P-*sacB-kan-rpsL*$^{+}$ // *ΔbgaA*::P$_{spxB}$-*itag-mNEONGreen-H3H4*	*CEP*::P-*sacB-kan-rpsL*$^{+}$ x NUS1782	Suc^{S} Kan^{R} Str^{S}	This study
NUS1861	*rpsL1 CEP*::P$_{spxB}$-*itag-popZ* // *ΔbgaA*::P$_{spxB}$-*itag-mNEONGreen-H3H4*	*CEP*::P$_{spxB}$-*itag-popZ* x NUS1836	Str^{R}	This study
NUS1257	*rpsL1 ΔbgaA*::P$_{spxB}$-*cpsC*	-	Str^{R}	(4)
NUS1783	*rpsL1 ΔbgaA*::P$_{spxB}$-*cpsC-L-sfgfp-H3H4*	*ΔbgaA*::P$_{spxB}$-*cpsC-L-sfgfp-H3H4* x NUS0121	Str^{R}	This study
NUS1837	*rpsL1 CEP*::P-*sacB-kan-rpsL*$^{+}$ // *ΔbgaA*::P$_{spxB}$-*cpsC-L-sfgfp-H3H4*	*CEP*::P-*sacB-kan-rpsL*$^{+}$ x NUS1837	Suc^{S} Kan^{R} Str^{S}	This study
NUS1862	*rpsL1 CEP*::P$_{spxB}$-*itag-popZ* // *ΔbgaA*::P$_{spxB}$-*cpsC-L-sfgfp-H3H4*	*CEP*::P$_{spxB}$-*itag-popZ* x NUS1837	Str^{R}	This study
NUS1890	*rpsL1 CEP*::P-*sacB-kan-rpsL*$^{+}$	*CEP*::P-*sacB-kan-rpsL*$^{+}$ x IU1781	Suc^{S} Kan^{R} Str^{S}	This study
NUS2335	*rpsL1 CEP*::P$_{spxB}$-*itag-popZ*	*CEP*::Ps$_{pxB}$-*itag-popZ* x NUS1890	Str^{R}	This study
NUS1891	*rpsL1 ΔcpsE* // *CEP*::P-*sacB-kan-rpsL*$^{+}$ // *ΔbgaA*::P$_{Zn}$-*cpsE*	*CEP*::P-*sacB-kan-rpsL*$^{+}$ x NUS0337	Suc^{S} Kan^{R} Str^{S}	This study
NUS2336	*rpsL1 ΔcpsE* // *CEP*::P$_{spxB}$-*itag-popZ* // *ΔbgaA*::P$_{Zn}$-*cpsE*	*CEP*::P$_{spxB}$-*itag-popZ* x NUS1891	Str^{R}	This study
NUS2333	*rpsL1 cpsC-H3H4*	*cpsC-H3H4* x NUS0422	Str^{R}	This study
NUS1996	*rpsL1 cpsC-H3H4* // *CEP*::P-*sacB-kan-rpsL*$^{+}$	*CEP*::P-*sacB-kan-rpsL*$^{+}$ x NUS2333	Suc^{S} Kan^{R} Str^{S}	This study
NUS2029	*rpsL1 cpsC-H3H4* // *CEP*::P$_{spxB}$-*itag-popZ*	*CEP*:: P$_{spxB}$-*itag-popZ* x NUS1996	Str^{R}	This study
NUS2334	*rpsL1 ΔcpsE cpsC-H3H4* // *ΔbgaA*::P$_{Zn}$-*cpsE*	*cpsC-H3H4* x NUS0426	Str^{R}	This study
NUS2030	*rpsL1 cpsC-H3H4 ΔcpsE* // *CEP*::P-*sacB-kan-rpsL*$^{+}$ // *ΔbgaA*::P$_{Zn}$-*cpsE*	*CEP*::P-*sacB-kan-rpsL*$^{+}$ x NUS2030	Suc^{S} Kan^{R} Str^{S}	This study
NUS3486	*rpsL1 cpsC-H3H4 ΔcpsE* // *CEP*::P$_{spxB}$-*itag-popZ* // *ΔbgaA*::P$_{Zn}$-*cpsE*	*CEP*::P$_{spxB}$-*itag-popZ* x NUS2030	Str^{R}	This study
NUS3863	*rpsL1 ΔcpsE cpsC-H3H4 cpsJ-P-sacB-kan-rpsL* // *ΔbgaA*::P$_{Zn}$-*cpsE*	*cpsJ-P-sacB-kan-rpsL* x NUS2334	Suc^{S} Kan^{R} Str^{S}	This study

NUS3893	*rpsL1* Δ*cpsE cpsC-H3H4 cpsJ-L-*$FLAG_3$ // Δ*bgaA*::P_{Zn}-*cpsE*	*cpsJ-L-*$FLAG_3$ x NUS3863	Str^R	This study
NUS4000	*rpsL1* Δ*cpsE cpsC-H3H4 cpsJ-L-*$FLAG_3$ // Δ*bgaA*::P_{Zn}-*cpsE* // *CEP*::P_{spxB}-*itag-popZ-P-spec*	*CEP*::P_{spxB}-*itag-popZ-P-spec* x NUS3893	Str^R $Spec^R$	This study

[a] Strains were constructed as described in the Methods session. "::" indicates an insertion into a gene. Unless otherwise specified, deletion mutants retain a 60bp region at the 5' and 3' of the corresponding open reading frames to avoid polarity.

[b] Primers used to synthesise fusion amplicons are listed in Tables S9. The amino acid sequence of the FLAG epitope is DYKDDDDK. $FLAG_3$ indicates three tandem FLAG epitopes. "L" is a linker (GSAGSAAGSG).

[c] Antibiotic resistance markers: Erm^R: erythromycin resistance, Kan^R: kanamycin resistance, Str^R, streptomycin resistance, Str^S, streptomycin sensitive, sucrose sensitive: Suc^S , spectinomycin resistance: $Spec^R$.

Supplemental Table 2. Oligonucleotides used in this study

Primer	Sequence (5' to 3')	Template	Amplified product
**For construction of NUS0882 (*cpsC-L-sfgfp*)**			
P91	GGTGATTGTGGCTATCCTTCA	D39	5' upstream of *cpsC* + 60bp of *cpsC*
P2088	CACCTGTGAACAGCTCTTCTCCTTTCATGCCAGAAC CAGCAGCGGAGCCAGCGGAACC		
P2087	ATGAAAGGAGAAGAGCTGTTCACAGG	IU9985	*sfgfp*
P319	CTACTTATAAAGCTCATCCATGCCGTG		
P2089	CTCACGGCATGGATGAGCTTTATAAGTAGGAGGAA GTTATGCCAACGTTAGAAATCTCAC	D39	60bp of *cpsC* + 3' downstre am of *cpsC*
P552	CTATTTTTTATTTTTCCCGTAATCTCC		
**For construction of NUS1613, NUS1611 and NUS0121 (Δ*bgaA*::P-*sacB-kan-rpsL*$^{+}$)**			
P86	GTTTGACTGCCGGTGTATCT	NUS0122	Δ*bgaA*::P-*sacB-kan-rpsL*$^{+}$
P6	TGCATGGTTACGATAGTCTTGG		
**For construction of NUS1624 and NUS1625 (Δ*bgaA*::P_{Zn}-*ftsZ*)**			
P86	GTTTGACTGCCGGTGTATCT	NUS0267	5' upstream of *bgaA* P_{Zn} + 60bp of *bgaA*
P1086	CAGCAGCTGTATCAAATGAAAATGTCATATTTCTCAT TCCTTTGTTATAATAGATTTATG		
P1084	ATGACATTTTCATTTGATACAGCTGCTG	D39	*ftsZ*
P1085	TTAACGATTTTTGAAAAATGGAGGTGTATCC		
P1087	GGATACACCTCCATTTTTCAAAAATCGTTAATTAGCT CTTCTAGGTTTGAGTGCAGG	D39	60bp of *bgaA* + 3' downstre am of *bgaA*
P6	TGCATGGTTACGATAGTCTTGG		
**For construction of NUS1625 and NUS1659 (Δ*ftsZ*::P-*kan-rpsL*$^{+}$)**			
P1088	GTGGATGGTTTCCAAGGGATTCGTGACCCACG	D39	5' upstream of *ftsZ* 60bp of *ftsZ*
P1089	CATTATCCATTAAAAATCAAACGGATCCTAGACACCA ATTACTTTAATCACTGCCCC		
P1	TAGGATCCGTTTGATTTTTAATGGATAATG	P-*kan-rpsL*$^{+}$ cassette	P-*kan-rpsL*$^{+}$
P2	GGGCCCCTTTCCTTATGCTTTTG		
P1090	CAAAAGCATAAGGAAAGGGGCCCGCCCCAATTTCA CAAGATGAAGATGAATTG	D39	60bp of *ftsZ* + 3' downstre am of *ftsZ*
P1091	GGCGTTGCAAGGGAGCAAGCACTGGTTTCACC		
**For construction of NUS NUS1706 (Δ*mapZ*::P-*kan-rpsL*$^{+}$)**			
P2701	GATTTCAAAAGCTAAATGTGTTAAGT	D39	5' upstream of *mapZ* 60bp of *mapZ*
P2702	TATCCATTAAAAATCAAACGGATCCTAAAAATCAAAT TGCGGTTC		
P1	TAGGATCCGTTTGATTTTTAATGGATAATG	P-*kan-rpsL*$^{+}$ cassette	P-*kan-rpsL*$^{+}$
P2	GGGCCCCTTTCCTTATGCTTTTG		
P2703	CAAAAGCATAAGGAAAGGGGCCCTGTAAGACAGGC TACTTTGTC	D39	60bp of *mapZ* + 3' downstre
P2704	CATGTACACGTTCTTCTTTGTAAG		

			am of *mapZ*
**For construction of NUS3474 (Δ*rocS*::P-*erm*)**			
O3090	GTCCTCCTTTGCTAGATG	D39	5' upstream of *rocS* + 60bp of *rocS*
O3091	CATTATCCATTAAAAATCAAACGGATCCTATGCTTGG CGAGATAATCCTAAG		
P1	TAGGATCCGTTTGATTTTTAATGGATAATG	P-*erm* cassette	P-*erm*
P2	GGGCCCCTTTCCTTATGCTTTTG		
O3092	CATTATCCATTAAAAATCAAACGGATCCTATGCTTGG CGAGATAATCCTAAG	D39	60bp of *rocS* + 3' downstre am of *rocS*
O3093	CCTATTTGAAAATAAGCAACCAG		
**For construction of NUS3476 (Δ*ccrZ::P-erm*)**			
O3086	GCAAGGCGACGTTGACG	D39	5' upstream of *ccrZ* + 60bp of *ccrZ*
O3087	CACATTATCCATTAAAAATCAAACGGATCCTAAGCCT TGCCACTTTTCC		
P1	TAGGATCCGTTTGATTTTTAATGGATAATG	P-*erm* cassette	P-*erm*
P2	GGGCCCCTTTCCTTATGCTTTTG		
O3088	CGTCCAAAAGCATAAGGAAAGGGGCCCGTCAATCG GGAGATTCATGG	D39	60bp of *ccrZ* + 3' downstre am of *ccrZ*
O3089	CAAAGGACGTTCCAAGCC		
**For construction of NUS3478 (Δ*pbp1A*::P-*erm*)**			
O1055	CAAGAAGGTCTTGCAAATGCC	D39	5' upstream of *pbp1A* 60bp of *pbp1A*
O1056	TCCATTAAAAATCAAACGGATCCTACAACAATCAAAT ACAACCCC		
P1	TAGGATCCGTTTGATTTTTAATGGATAATG	P-*erm* cassette	P-*erm*
P2	GGGCCCCTTTCCTTATGCTTTTG		
O1057	GGCGCAGAATCGTTGGTTTGTTCATCTTGTTTTACC ACCTAATAA	D39	60bp of *pbp1A* + 3' downstre am of *pbp1A*
O1058	CTGCCCCTTGTGTTCATAGCG		
**For construction of NUS3505 (Δ*pbp1B*::P-*erm*)**			
O1059	GTAAAATAATCAAGGCTATAG	D39	5' upstream of *pbp1B* + 60bp of *pbp1B*
O1060	CCATTAAAAATCAAACGGATCCTATCTAGCGATAGC AGTAACTC		
P1	TAGGATCCGTTTGATTTTTAATGGATAATG	P-*erm* cassette	P-*erm*
P2	GGGCCCCTTTCCTTATGCTTTTG		
O1061	CAAAAGCATAAGGAAAGGGGCCCTTGTTTTTGCTGG AAAAATTCC	D39	60bp of *pbp1B* + 3' downstre am of *pbp1B*
O1062	CAATTTGAAGCAGCGCCACAC		
**For construction of NUS3487 (Δ*pbp2A*::P-*erm*)**			
O1063	GTCTAACAGATTAGAAAACAC	D39	

O1064	CCATTAAAAATCAAACGGATCCTAGCGAAGATTAAG GAAAAGGC		5' upstream of *pbp2A* + 60bp of *pbp2A*
P1	TAGGATCCGTTTGATTTTTAATGGATAATG	P-*erm* cassette	P-*erm*
P2	GGGCCCCTTTCCTTATGCTTTTG		
O1065	CAAAAGCATAAGGAAAGGGGCCCTTCACTTGTTTCT TTTTTAAAA	D39	60bp of *pbp2A* + 3' downstream of *pbp2A*
O1066	GATAGAAAGCAAGGCTTTGTGG		
**For construction of NUS1858 (Δ*bgaA*::P$_{Zn}$-*ezrA*)**			
P86	GTTTGACTGCCGGTGTATCT	NUS0267	5' upstream of *bgaA* P$_{Zn}$ + 60bp of *bgaA*
P3087	ATAAATTAGTTGTCCATTAGACATATTTCTCATTCCTT TGTTATA		
P3078	ATGTCTAATGGACAACTAATTTATTTAATG	D39	*ezrA*
P3079	TTAAAAACGAATCGTTTCACG		
P3073	CACGTGAAACGATTCGTTTTTAATTAGCTCTTCTAGG TTTGAG	D39	60bp of *bgaA* + 3' downstream of *bgaA*
P6	TGCATGGTTACGATAGTCTTGG		
**For construction of NUS1869 (Δ*ezrA*::P-*kan-rpsL*$^+$)**			
P3072	CCAAATCCACAGTTTGAAGGAC	D39	5' upstream of *ezrA* + 60bp of *ezrA*
P3073	CCATTAAAAATCAAACGGATCCTAAGCCAGAACTAA AATGACTGC		
P1	TAGGATCCGTTTGATTTTTAATGGATAATG	P-*kan-rpsL*$^+$ cassette	P-*kan-rpsL*$^+$
**P2**	**GGGCCCCTTTCCTTATGCTTTTG**		
P3075	CAAAAGCATAAGGAAAGGGGCCCGGTGTAACCAAT CGCTTTG	D39	60bp of *ezrA* + 3' downstream of *ezrA*
P3076	CGTGGCATAATATTTCGCCAACC		
**For construction of NUS1674 (Δ*bgaA*::P$_{Zn}$-*ftsX*)**			
P86	GTTTGACTGCCGGTGTATCT	NUS0267	5' upstream of *bgaA* P$_{Zn}$ + 60bp of *bgaA*
P2338	CAAATAAATGGCGAAAAAATCTACTAATCATATTTCT CATTCCTTTGTTATAATAG		
P2340	ATGATTAGTAGATTTTTTCGCCATTTATTTG	D39	*ftsX*
P2341	TAAATCTTCAAGAATCGGCGCATGG		
P2339	GGAATATCCATGCGCCGATTCTTGAAGATTTATTAG CTCTTCTAGGTTTGAGTGCAGG	D39	60bp of *bgaA* + 3' downstream of *bgaA*
P6	TGCATGGTTACGATAGTCTTGG		
**For construction of NUS1675 and NUS1679 (Δ*bgaA*::P$_{Zn}$-*ftsQ*)**			
P86	GTTTGACTGCCGGTGTATCT	NUS0267	5' upstream of *bgaA*
P2342	CCTCATTTTTCTTATCTTTTGACATATTTCTCATTCCT TTGTTATAATAGATTTATG		

			P_{Zn} + 60bp of *bgaA*
P2344	ATGTCAAAAGATAAGAAAAATGAG	D39	*ftsQ*
P2345	CTAGCGACGCGATGAACGCTG		
P2343	CAAACAAATCAGCGTTCATCGCGTCGCTAGTTAGCT CTTCTAGGTTTGAGTGCAGG	D39	60bp of *bgaA* + 3' downstream of *bgaA*
P6	TGCATGGTTACGATAGTCTTGG		
**For construction of NUS1676 and NUS1680 (△*bgaA*::P_{Zn}-*pbp2X*)**			
P86	GTTTGACTGCCGGTGTATCT	NUS0267	5' upstream of *bgaA* P_{Zn} + 60bp of *bgaA*
P2346	CGGATTACTCTTTTTGTCCACTTCATATTTCTCATTC CTTTGTTATAATAGATTTATG		
**P2348**	ATGAAGTGGACAAAAAGAGTAATCC	D39	*pbp2X*
P2349	TTAGTCTCCTAAAGTTAATGTAATTTTTTTAATG		
P2347	CATTAAAAAAATTACATTAACTTTAGGAGACTAATTA GCTCTTCTAGGTTTGAGTGCAGG	D39	60bp of *bgaA* + 3' downstream of *bgaA*
P6	TGCATGGTTACGATAGTCTTGG		
**For construction of NUS1677 (△*bgaA*::P_{Zn}-*pbp2B*)**			
P86	GTTTGACTGCCGGTGTATCT	NUS0267	5' upstream of *bgaA* P_{Zn} + 60bp of *bgaA*
P2350	GGCTGTTAAATTTTCTCATACAAATCAGTCTCATATT TCTCATTCCTTTGTTATAATAG		
P2352	ATGAGACTGATTTGTATGAGAAAATTTAAC	D39	*pbp2B*
P2353	CTAATTCATTGGATGGTATTTTTG		
P2351	CTGTATCAAAAATACCATCCAATGAATTAGTTAGCTC TTCTAGGTTTGAGTGCAGG	D39	60bp of *bgaA* + 3' downstream of *bgaA*
P6	TGCATGGTTACGATAGTCTTGG		
**For construction of NUS1810 and NUS1860 (△*bgaA*::P_{Zn}-*ftsW*)**			
P86	GTTTGACTGCCGGTGTATCT	NUS0267	5' upstream of *bgaA* P_{Zn} + 60bp of *bgaA*
P3005	AATAAGTGCCTCTTACTAATCTTCATATTTCTCATTC CTTTGTTA		
P3007	ATGAAGATTAGTAAGAGGCAC	D39	*ftsW*
P3008	CTACTTCAACAGAAGGTTCATTGG		
P3006	CCAATGAACCTTCTGTTGAAGTAGTTAGCTCTTCTA GGTTTGAG	D39	60bp of *bgaA* + 3' downstream of *bgaA*
P6	TGCATGGTTACGATAGTCTTGG		
**For construction of NUS1749 (△*ftsX*::P-*kan-rpsL*$^{+}$)**			
P2358	GCAGGCTAAGCTCTATCAAATGG	D39	5' upstream of *ftsX* + 60bp of *ftsX*
P2359	CACATTATCCATTAAAAATCAAACGGATCCTAACCAT TTCGTTTCAAACTTTTTAAGGC		
P1	TAGGATCCGTTTGATTTTTAATGGATAATG		

P2	GGGCCCCTTTCCTTATGCTTTTG	P-*kan-rpsL*+ cassette	P-*kan-rpsL*+
P2360	GTACTAAACGTCCAAAAGCATAAGGAAAGGGGCCC TGGGGTTTTCATTGGTTCATTGGG	D39	60bp of *ftsX* + 3' downstream of *ftsX*
P2361	GCTGTGTAGTTCATTGGGATAGTC		
**For construction of NUS1750 and NUS2068 (Δ*ftsQ*::P-*kan-rpsL*+)**			
**P2354**	CCCAAGTTCATCGAAGATGGTTGG	D39	5' upstream of *ftsQ* + 60bp of *ftsQ*
**P2355**	CACATTATCCATTAAAAATCAAACGGATCCTATGATA ACTCTTTCAATTCTTCGAGGG		
P1	TAGGATCCGTTTGATTTTTAATGGATAATG	P-*kan-rpsL*+ cassette	P-*kan-rpsL*+
P2	GGGCCCCTTTCCTTATGCTTTTG		
P2356	CTAAACGTCCAAAAGCATAAGGAAAGGGGCCCAAA AAACAAGAGGAAGAGAGCAATCG	D39	60bp of *ftsQ* + 3' downstream of *ftsQ*
P2357	cgttgcagatataataacaacaagc		
**For construction of NUS1751 and NUS2185 (Δ*pbp2X*::P-*kan-rpsL*+)**			
P2362	CATTGAGAAGGGAATGGTGACC	D39	5' upstream of *pbp2X* + 60bp of *pbp2X*
P2363	CACATTATCCATTAAAAATCAAACGGATCCTATTCAG CCGGCGATTTCCGATTTTTGGTC		
P1	TAGGATCCGTTTGATTTTTAATGGATAATG	P-*kan-rpsL*+ cassette	P-*kan-rpsL*+
P2	GGGCCCCTTTCCTTATGCTTTTG		
P2364	CTAAACGTCCAAAAGCATAAGGAAAGGGGCCCGTT CGTGCTAACACAGCTATCAAGGAC	D39	60bp of *pbp2X* + 3' downstream of *pbp2X*
P2365	CTAGTTTGTTCTTTGATTTTCATTG		
**For construction of NUS1775 (Δ*pbp2B*::P-*kan-rpsL*+)**			
P2366	CTATTAAAGATTTTTTCCTTGCTTTCGCG	D39	5' upstream of *pbp2B* + 60bp of *pbp2B*
P2367	CATTATCCATTAAAAATCAAACGGATCCTATAAATTA AGCCGAATCGGAATCGAATGGC		
P1	TAGGATCCGTTTGATTTTTAATGGATAATG	P-*kan-rpsL*+ cassette	P-*kan-rpsL*+
P2	GGGCCCCTTTCCTTATGCTTTTG		
P2368	CAAAAGCATAAGGAAAGGGGCCCGGACCTTCCATT GCGCGTGACATTATCAATCTG	D39	60bp of *pbp2B* + 3' downstream of *pbp2B*
P2369	GTAGATAGCACTTGGTGCAACTTTC		
**For construction of NUS1826 and NUS2184 (Δ*ftsW*::P-*kan-rpsL*+)**			
P3013	GTATTGGTAACTCTCTCAAAC	D39	5' upstream of *ftsW* + 60bp of *ftsW*
P3014	CCATTAAAAATCAAACGGATCCTATAAAAGCAAGTA GGGAATC		
P1	TAGGATCCGTTTGATTTTTAATGGATAATG		

P2	GGGCCCCTTTCCTTATGCTTTTG	P-*kan-rpsL*$^+$ cassette	P-*kan-rpsL*$^+$
P3015	CCAAAAGCATAAGGAAAGGGGCCCAAACGCGCTAA GTTGTACCG	D39	60bp of *ftsW* + 3' downstream of *ftsW*
P3016	CATCCTCAATCAGATAGGTTTTAG		
**For construction of NUS1704 (∆*khpA*::P-*sacB-kan-rpsL*$^+$)**			
P2695	GGGGCATCAATGCCATAAGTGTACTCAAAG	D39	5' upstream of *khpA* + 60bp of *khpA*
P2696	CATTATCCATTAAAAATCAAACGGATCCTACGCAATA ATGAGATT		
P249	TAGGATCCGTTTGATTTTTAATGGATAATGTTAAGGA TCGATCCGTTTGATTTTTAATGG	P-*sacB-kan-rpsL*$^+$ cassette	P-*sacB-kan-rpsL*$^+$
P250	GGGCCCCTTTCCTTATGCTTTTGGACGTTTAGTACC GTATTTAGAACGG		
P2697	CAAAAGCATAAGGAAAGGGGCCCATTGTCTACTCTG TCCCAACTG	D39	60bp of *khpA* + 3' downstream of *khpA*
P2698	GTCATGAATCGGACTCCTACCTGTGTTCGG		
**For construction of NUS1717 (∆*khpA*)**			
P2695	GGGGCATCAATGCCATAAGTGTACTCAAAG	D39	5' upstream of *khpA* + 60bp of *khpA*
P2699	GTATTCAGTTGGGACAGAGTAGACAATCGCAATAAT GAGATTTTC		
P2700	GAAAATCTCATTATTGCGATTGTCTACTCTGTCCCAA CTGAATAC	D39	60bp of *khpA* + 3' downstream of *khpA*
P2698	GTCATGAATCGGACTCCTACCTGTGTTCGG		
**For construction of NUS1740 (∆*gpsB*::P-*kan-rpsL*$^+$)**			
P2674	CCTAAGACAATGGCTGCGATA	D39	5' upstream of *gpsB* + 60bp of *gpsB*
P2675	CCATTAAAAATCAAACGGATCCTAGACTTCACGTCC AAACTCTTGTTC		
P1	TAGGATCCGTTTGATTTTTAATGGATAATG	P-*kan-rpsL*$^+$ cassette	P-*kan-rpsL*$^+$
P2	GGGCCCCTTTCCTTATGCTTTTG		
P2676	CAAAAGCATAAGGAAAGGGGCCCCTGAATAGATTG GAAAAAGAAGTTTTTGG	D39	60bp of *gpsB* + 3' downstream of *gpsB*
P2677	TTGAGGAATGGAACTGGATCAG		
**For construction of NUS1748 (∆*mreC*::P-*kan-rpsL*$^+$)**			
P2278	GGATTCGATTGTTCACCGATTG	D39	5' upstream of *mreC* + 60bp of *mreC*
P2279	TTAAAAATCAAACGGATCCTACACAAGCAGAACAGT GACAAAAAC		
P1	TAGGATCCGTTTGATTTTTAATGGATAATG	P-*kan-rpsL*$^+$ cassette	P-*kan-rpsL*$^+$
P2	GGGCCCCTTTCCTTATGCTTTTG		

P2280	CAAAAGCATAAGGAAAGGGGCCCTTAAATTGAGTGC AGATACTC	D39	60bp of *mreC* + 3' downstream of *mreC*
P2281	GCTGATACTTGCTCCTGAATTTG		
**For construction of NUS1753 (Δ*pbp2B*::P-*kan-rpsL*⁺)**			
P2366	CTATTAAAGATTTTTTCCTTGCTTTCGCG	D39	5' upstream of *pbp2B* + 60bp of *pbp2B*
P2367	CATTATCCATTAAAAATCAAACGGATCCTATAAATTA AGCCGAATCGGAATCGAATGGC		
P1	TAGGATCCGTTTGATTTTTAATGGATAATG	P-*kan-rpsL*⁺ cassette	P-*kan-rpsL*⁺
P2	GGGCCCCTTTCCTTATGCTTTTG		
P2368	CATTATCCATTAAAAATCAAACGGATCCTATAAATTA AGCCGAATCGGAATCGAATGGC	D39	60bp of *pbp2B* + 3' downstream of *pbp2B*
P2369	GTAGATAGCACTTGGTGCAACTTTC		
**For construction of NUS1806 (Δ*rodA*::P-*kan-rpsL*⁺)**			
P3009	GATAAGGCTTTAGTAGGAGAAG	D39	5' upstream of *rodA* + 60bp of *rodA*
P3010	CATTAAAAATCAAACGGATCCTATAGAAAAAATACTG GCAAGAGC		
P1	TAGGATCCGTTTGATTTTTAATGGATAATG	P-*kan-rpsL*⁺ cassette	P-*kan-rpsL*⁺
P2	GGGCCCCTTTCCTTATGCTTTTG		
P3011	CCAAAAGCATAAGGAAAGGGGCCCGAAAAGAGCGG AAAAGTCCC	D39	60bp of *rodA* + 3' downstream of *rodA*
P3012	CTGTAAAATAAGCTGCTATAG		
**For construction of NUS0422 (Δ*cpsCDE*::P-*sacB-kan-rpsL*⁺)**			
P91	GGTGATTGTGGCTATCCTTCA	D39	5' upstream of *cpsC* + 60bp of *cpsC*
P422	CATTATCCATTAAAAATCAAACGGATCCTATTTCCAC AAGGTTTTAAATAATTGAAATAC		
P249	TAGGATCCGTTTGATTTTTAATGGATAATGTTAAGGA TCGATCCGTTTGATTTTTAATGG	P-*sacB-kan-rpsL*⁺ cassette	P-*sacB-kan-rpsL*⁺
P250	GGGCCCCTTTCCTTATGCTTTTGGACGTTTAGTACC GTATTTAGAACGG		
P252	CAAAAGCATAAGGAAAGGGGCCCGACATTGAAATTT TATTGAAGACAGTTAAAG	D39	60bp of *cpsE* + 3' downstream of *cpsE*
P65	TCGTCCCACCACTAGATAATAGCC		
**For construction of NUS1571 (*cpsC-L-FLAG*$_3$)**			
P91	GGTGATTGTGGCTATCCTTCA	D39	5' upstream of *cpsC* + *cpsC* with C-terminus *FLAG*$_3$
P1759	TTTATCATCATCATCTTTATAATCTTTATCATCATCAT CTTTATAATCTTTATCATCATCATCTTTATAATCGCCA GAACCAGCAGCGGAGCCAGCGGAACCTTTCATTTT GTCCAAATC		
P1633	GATAAAGATTATAAAGATGATGATGATAAATAGGAG GAAGTTATGCCAACGTTAGAAATC	D39	*FLAG*$_3$ + *cpsD* +

P65	TCGTCCCACCACTAGATAATAGCC		*cpsE* + 3' downstream of *cpsE*
**For construction of NUS1915, NUS1914 and NUS1918 (*ftsZ*⁺-P-*kan-rpsL*⁺)**			
P1080	AGTGGTGCCGATATGGTCTTCATCACTGCT	D39	5' upstream of *ftsZ* + *ftsZ*
P1081	CATTATCCATTAAAAATCAAACGGATCCTATTAACGATTTTTGAAAAATGGAGGTGTATC		
P1	TAGGATCCGTTTGATTTTTAATGGATAATG	P-*kan-rpsL*⁺ cassette	P-*kan-rpsL*⁺
P2	GGGCCCCTTTCCTTATGCTTTTG		
P1082	CAAAAGCATAAGGAAAGGGGCCCGCCCCAATTTCACAAGATGAAGATG	D39	3' downstream of *ftsZ*
P1083	TCATTGGGAGAGCCGGTTCCTGTGAAGAAT		
**For construction of NUS2034, NUS2033 and NUS2037 (*ftsZ-L-sfgfp*)**			
P1080	AGTGGTGCCGATATGGTCTTCATCACTGCT	IU9985	5' upstream of *ftsZ* + ftsZ-L-sfgfp + 3' downstream of *ftsZ*
P1083	TCATTGGGAGAGCCGGTTCCTGTGAAGAAT		
**For construction of NUS2235, NUS0546 and NUS3385 (Δ*cpsA*::P-*sacB-kan-rpsL*⁺)**			
P9	GGTGAGTAGGGAAGAAGAGGTA	D39	5' upstream of *cpsA* + 60bp of *cpsA*
P206	CATTATCCATTAAAAATCAAACGGATCCTAAACTATATTAACACTTCGCTTCACTTTC		
P249	TAGGATCCGTTTGATTTTTAATGGATAATGTTAAGGATCGATCCGTTTGATTTTTAATGG	P-*sacB-kan-rpsL*⁺ cassette	P-*sacB-kan-rpsL*⁺
P250	GGGCCCCTTTCCTTATGCTTTTGGACGTTTAGTACCGTATTTAGAACGG		
P207	CAAAAGCATAAGGAAAGGGGCCCGATGATAGTAGTTTAGCTGTAGTTAAAGCAGCTATAC	D39	60bp of *cpsA* + 3' downstream of *cpsA*
P428	CTATTTCATTTTGTCCAAATC		
**For construction of NUS2296 (Δ*cpsA*::P-*sacB-kan-rpsL*⁺)**			
P9	GGTGAGTAGGGAAGAAGAGGTA	D39	5' upstream of *cpsA* + 60bp of *cpsA*
P206	CATTATCCATTAAAAATCAAACGGATCCTAAACTATATTAACACTTCGCTTCACTTTC		
P249	TAGGATCCGTTTGATTTTTAATGGATAATGTTAAGGATCGATCCGTTTGATTTTTAATGG	P-*sacB-kan-rpsL*⁺ cassette	P-*sacB-kan-rpsL*⁺
P250	GGGCCCCTTTCCTTATGCTTTTGGACGTTTAGTACCGTATTTAGAACGG		
P207	CAAAAGCATAAGGAAAGGGGCCCGATGATAGTAGTTTAGCTGTAGTTAAAGCAGCTATAC	NUS0679	60bp of *cpsA* + Δ*cpsC* + 3' downstream of *cpsC*
P552	CTATTTCATTTTGTCCAAATC		
**For construction of NUS0734 (*cpsA-L-FLAG₃*)**			
P9	GGTGAGTAGGGAAGAAGAGGTA	D39	5' upstream of *cpsA* +
O1767	TTTATCATCATCATCTTTATAATCTTTATCATCATCATCTTTATAATCTTTATCATCATCATCTTTATAATCGCCA		

	GAACCAGCAGCGGAGCCAGCGGAACCTCTACCCTC CATCACATCCTG		*cpsA* with C-terminus $FLAG_3$
O1768	GATTATAAAGATGATGATGATAAATAGAATGATAGAC GTCCATTC	D39	$FLAG_3$ + 3' downstream of *cpsA*
P552	CTATTTCATTTTGTCCAAATC		
**For construction of NUS0734 (*cpsA-L-$FLAG_3$*)**			
P9	GGTGAGTAGGGAAGAAGAGGTA	D39	5' upstream of *cpsA* + *cpsA* with C-terminus $FLAG_3$
O1767	TTTATCATCATCATCTTTATAATCTTTATCATCATCAT CTTTATAATCTTTATCATCATCATCTTTATAATCGCCA GAACCAGCAGCGGAGCCAGCGGAACCTCTACCCTC CATCACATCCTG		
O1768	GATTATAAAGATGATGATGATAAATAGAATGATAGAC GTCCATTC	NUS0679	$FLAG_3$ + Δ*cpsC* +3' downstream of *cpsC*
P552	CTATTTCATTTTGTCCAAATC		
**For construction of NUS0713, NUS2302, NUS2647 and NUS3863 (Δ*cpsJ*::P-*sacB-kan-rpsL*$^+$)**			
P51	GAACATGGAAATGTGGAAGATGAG	D39	5' upstream of *cpsJ* + 60bp of *cpsJ*
P52	CATTATCCATTAAAAATCAAACGGATCCTAAAAGTTG CTTAAAGTTAGTAATCC		
P249	TAGGATCCGTTTGATTTTTAATGGATAATGTTAAGGA TCGATCCGTTTGATTTTTAATGG	P-*sacB-kan-rpsL*$^+$ cassette	P-*sacB-kan-rpsL*$^+$
P250	GGGCCCCTTTCCTTATGCTTTTGGACGTTTAGTACC GTATTTAGAACGG		
P53	CAAAAGCATAAGGAAAGGGGCCCATGTTATTTTATA AAGAAATAAAGAGTATTATTGG	D39	60bp of *cpsJ* + 3' downstream of *cpsJ*
P54	CATAGCCGAAGGAAGGATTGT		
**For construction of NUS3063, NUS3065 and NUS3893 (*cpsJ-L-$FLAG_3$*)**			
P51	GAACATGGAAATGTGGAAGATGAG	D39	5' upstream of *cpsJ* + *cpsJ* with C-terminus $FLAG_3$
O1665	CTTTATAATCTTTATCATCATCATCTTTATAATCGCCA GAACCAGCAGCGGAGCCAGCGGAACCTGTTAGAAA CTTTTTTAATTC		
O1666	GATGATGATAAAGATTATAAAGATGATGATGATAAAG ATTATAAAGATGATGATGATAAATAAGAACCAATAAG TACGAG	D39	$FLAG_3$ + 3' downstream of *cpsJ*
P552	CTATTTCATTTTGTCCAAATC		
**For construction of NUS1137, NUS1327, NUS2648, NUS3283 and NUS3291 (Δ*cpsH*::P-*sacB-kan-rpsL*$^+$)**			
P43	AAGGACATGATGTGGTTTGTTATG	D39	5' upstream of *cpsH* + 60bp of *cpsH*
P44	CATTATCCATTAAAAATCAAACGGATCCTACAAAAAA AATAAAATAACAATAACAGG		

P249	TAGGATCCGTTTGATTTTTAATGGATAATGTTAAGGATCGATCCGTTTGATTTTTAATGG	P-*sacB*-*kan*-*rpsL*$^+$ cassette	P-*sacB*-*kan*-*rpsL*$^+$
P250	GGGCCCCTTTCCTTATGCTTTTGGACGTTTAGTACCGTATTTAGAACGG		
P45	CAAAAGCATAAGGAAAGGGGCCCTTTTACGTGGTTATTATCTGTGCATTTATAGG	D39	60bp of *cpsH* + 3' downstream of *cpsH*
P46	CTCCAGACAACATTCCCTCAA		
**For construction of NUS3064 and NUS3103 (*cpsH-L-FLAG*$_3$)**			
P43	AAGGACATGATGTGGTTTGTTATG	D39	5' upstream of *cpsH* + *cpsH* with C-terminus *FLAG*$_3$
O1780	TTTATCATCATCATCTTTATAATCTTTATCATCATCATCTTTATAATCTTTATCATCATCATCTTTATAATCGCCAGAACCAGCAGCGGAGCCAGCGGAACCTTTTTCTTGCTTAGTCAATCTC		
O1781	GATTATAAAGATGATGATGATAAATAAAAATGGATGGGGAAATTC	D39	*FLAG*$_3$ + 3' downstream of *cpsH*
P46	CTCCAGACAACATTCCCTCAA		
**For construction of NUS2236 (∆*cpsB*::P-*sacB-kan-rpsL*$^+$)**			
P9	GGTGAGTAGGGAAGAAGAGGTA	D39	5' upstream of *cpsB* + 60bp of *cpsB*
P731	CATTATCCATTAAAAATCAAACGGATCCTACTCTCTTGACTTGGGACCGTCATCTACATC		
P249	TAGGATCCGTTTGATTTTTAATGGATAATGTTAAGGATCGATCCGTTTGATTTTTAATGG	P-*sacB*-*kan*-*rpsL*$^+$ cassette	P-*sacB*-*kan*-*rpsL*$^+$
P250	GGGCCCCTTTCCTTATGCTTTTGGACGTTTAGTACCGTATTTAGAACGG		
P732	CAAAAGCATAAGGAAAGGGGCCCAAGGCTCAGGAACTTTTTATAGACAATCCTCG	NUS1571	60bp of *cpsB* + *cpsC-L-FLAG*$_3$ + 3' downstream of *cpsC*
P552	CTATTTTTTATTTTTCCCGTAATCTCC		
**For construction of NUS3286 (∆*cpsB*::P-*sacB-kan-rpsL*$^+$)**			
P9	GGTGAGTAGGGAAGAAGAGGTA	D39	5' upstream of *cpsB* + 60bp of *cpsB*
P731	CATTATCCATTAAAAATCAAACGGATCCTACTCTCTTGACTTGGGACCGTCATCTACATC		
P249	TAGGATCCGTTTGATTTTTAATGGATAATGTTAAGGATCGATCCGTTTGATTTTTAATGG	P-*sacB*-*kan*-*rpsL*$^+$ cassette	P-*sacB*-*kan*-*rpsL*$^+$
P250	GGGCCCCTTTCCTTATGCTTTTGGACGTTTAGTACCGTATTTAGAACGG		
P732	CAAAAGCATAAGGAAAGGGGCCCAAGGCTCAGGAACTTTTTATAGACAATCCTCG	D39	60bp of *cpsB* + 3' downstream of *cpsB*
P552	CTATTTTTTATTTTTCCCGTAATCTCC		
**For construction of NUS3294 (∆*cpsB*::P-*erm*)**			
P9	GGTGAGTAGGGAAGAAGAGGTA	D39	5' upstream of *cpsB* +
P731	CATTATCCATTAAAAATCAAACGGATCCTACTCTCTTGACTTGGGACCGTCATCTACATC		

			60bp of *cpsB*
P1	TAGGATCCGTTTGATTTTTAATGGATAATG	*P-erm* cassette	P-*erm*
P2	GGGCCCCTTTCCTTATGCTTTTG		
P732	CAAAAGCATAAGGAAAGGGGCCCAAGGCTCAGGAA CTTTTTATAGACAATCCTCG	D39	60bp of *cpsB* + 3' downstre am of *cpsB*
P552	CTATTTTTTATTTTTCCCGTAATCTCC		
**For construction of NUS3325 (∆*cpsB*::P-*erm*)**			
P9	GGTGAGTAGGGAAGAAGAGGTA	NUS0734	5' upstream of *cpsA* + *cpsA-L-FLAG$_3$* + + 60bp of *cpsB*
P731	CATTATCCATTAAAAATCAAACGGATCCTACTCTCTT GACTTGGGACCGTCATCTACATC		
P1	TAGGATCCGTTTGATTTTTAATGGATAATG	*P-erm* cassette	P-*erm*
P2	GGGCCCCTTTCCTTATGCTTTTG		
P732	CAAAAGCATAAGGAAAGGGGCCCAAGGCTCAGGAA CTTTTTATAGACAATCCTCG	D39	60bp of *cpsB* + 3' downstre am of *cpsB*
P552	CTATTTTTTATTTTTCCCGTAATCTCC		
**For construction of NUS2307 (∆*cpsD*::P-*sacB-kan-rpsL*$^+$)**			
P91	GGTGATTGTGGCTATCCTTCA	NUS1571	5' upstream of *cpsC* + *cpsC-L-FLAG$_3$* + 60bp of *cpsD*
P788	CATTATCCATTAAAAATCAAACGGATCCTAATATTCC TCTGCCTTTTTTACAAAATCC		
P249	TAGGATCCGTTTGATTTTTAATGGATAATGTTAAGGA TCGATCCGTTTGATTTTTAATGG	P-*sacB-kan-rpsL*$^+$ cassette	P-*sacB-kan-rpsL*$^+$
P250	GGGCCCCTTTCCTTATGCTTTTGGACGTTTAGTACC GTATTTAGAACGG		
P425	CCAAAAGCATAAGGAAAGGGGCCCTTCGATACTTCA GTAGACAAATACGGTTCTTATGG	NUS0267	60bp of *cpsD* + 3' downstre am of *cpsD*
P65			
**For construction of NUS3279, NUS3287 and NUS3295 (∆*cpsD*::P-*sacB-kan-rpsL*$^+$)**			
P91	GGTGATTGTGGCTATCCTTCA	D39	
P788	CATTATCCATTAAAAATCAAACGGATCCTAATATTCC TCTGCCTTTTTTACAAAATCC		
P249	TAGGATCCGTTTGATTTTTAATGGATAATGTTAAGGA TCGATCCGTTTGATTTTTAATGG	P-*sacB-kan-rpsL*$^+$ cassette	P-*sacB-kan-rpsL*$^+$
P250	GGGCCCCTTTCCTTATGCTTTTGGACGTTTAGTACC GTATTTAGAACGG		
P425	CCAAAAGCATAAGGAAAGGGGCCCTTCGATACTTCA GTAGACAAATACGGTTCTTATGG	D39	
P65			
**For construction of NUS2238 (∆*cpsG*::P-*sacB-kan-rpsL*$^+$)**			
P1112	GACTTTGTTCTCATTACAAATGTGGAACAG	D39	5' upstream of *cpsG* + 60bp of *cpsG*
P1113	CATTATCCATTAAAAATCAAACGGATCCTAATAGATA ACTCTTATTCCACCTGTCATTC		

P249	TAGGATCCGTTTGATTTTTAATGGATAATGTTAAGGA TCGATCCGTTTGATTTTTAATGG	P-*sacB-kan-rpsL*$^+$ cassette	P-*sacB-kan-rpsL*$^+$
P250	GGGCCCCTTTCCTTATGCTTTTGGACGTTTAGTACC GTATTTAGAACGG		
P1114	CAAAAGCATAAGGAAAGGGGCCCAACTCTTGTTTAG AATTTGAGAAGATTATATCAGGGG	D39	60bp of *cpsG* + 3' downstream of *cpsG*
P1115	CCTTTGTTTACAAGCTCATCTTCCACATTTCC		
**For construction of NUS3375 and NUS3384 (∆*cpsG*::*P-erm*)**			
P1112	GACTTTGTTCTCATTACAAATGTGGAACAG	D39	5' upstream of *cpsG* + 60bp of *cpsG*
P1113	CATTATCCATTAAAAATCAAACGGATCCTAATAGATA ACTCTTATTCCACCTGTCATTC		
P1	TAGGATCCGTTTGATTTTTAATGGATAATG	*P-erm* cassette	P-*erm*
P2	GGGCCCCTTTCCTTATGCTTTTG		
P1114	CAAAAGCATAAGGAAAGGGGCCCAACTCTTGTTTAG AATTTGAGAAGATTATATCAGGGG	D39	60bp of *cpsG* + 3' downstream of *cpsG*
P1115	CCTTTGTTTACAAGCTCATCTTCCACATTTCC		
**For construction of NUS3375 and NUS3384 (∆*cpsG*::*P-erm*)**			
P1112	GACTTTGTTCTCATTACAAATGTGGAACAG	D39	5' upstream of *cpsG* + 60bp of *cpsG*
P1113	CATTATCCATTAAAAATCAAACGGATCCTAATAGATA ACTCTTATTCCACCTGTCATTC		
P1	TAGGATCCGTTTGATTTTTAATGGATAATG	*P-erm* cassette	P-*erm*
P2	GGGCCCCTTTCCTTATGCTTTTG		
P1114	CAAAAGCATAAGGAAAGGGGCCCAACTCTTGTTTAG AATTTGAGAAGATTATATCAGGGG	NUS3064	60bp of *cpsG* + *cpsH-L-FLAG*$_3$ + 3' downstream of *cpsH*
P46	CCTTTGTTTACAAGCTCATCTTCCACATTTCC		
**For construction of NUS2239 (∆*cpsI*::P-*sacB-kan-rpsL*$^+$)**			
P1116	CATTATCCATTAAAAATCAAACGGATCCTACCGGGT AGCTCCCGATGATATATCAGATG	D39	5' upstream of *cpsI* + 60bp of *cpsI*
P1117	CATTATCCATTAAAAATCAAACGGATCCTACCGGGT AGCTCCCGATGATATATCAGATG		
P249	TAGGATCCGTTTGATTTTTAATGGATAATGTTAAGGA TCGATCCGTTTGATTTTTAATGG	P-*sacB-kan-rpsL*$^+$ cassette	P-*sacB-kan-rpsL*$^+$
P250	GGGCCCCTTTCCTTATGCTTTTGGACGTTTAGTACC GTATTTAGAACGG		
P1118	CAAAAGCATAAGGAAAGGGGCCCTTTTCAGATAAAA ATAATGCCATGCAGATCATGCAAC	D39	60bp of *cpsI* + 3' downstream of *cpsI*
P1119	GACCAACATATCCCGTACCTGCTACTGC		
**For construction of NUS3326 (∆*cpsI*::*P-erm*)**			
P1116	CATTATCCATTAAAAATCAAACGGATCCTACCGGGT AGCTCCCGATGATATATCAGATG	D39	5' upstream of *cpsI* + 60bp of *cpsI*
P1117	CATTATCCATTAAAAATCAAACGGATCCTACCGGGT AGCTCCCGATGATATATCAGATG		

P1	TAGGATCCGTTTGATTTTTAATGGATAATG	*P-erm* cassette	P-*erm*
P2	GGGCCCCTTTCCTTATGCTTTTG		
P1118	CAAAAGCATAAGGAAAGGGGCCCTTTTCAGATAAAA ATAATGCCATGCAGATCATGCAAC	D39	60bp of *cpsI* + 3' downstre am of *cpsI*
P1119	GACCAACATATCCCGTACCTGCTACTGC		
**For construction of NUS3378 (Δ*cpsI*::*P-erm*)**			
P51	CATTATCCATTAAAAATCAAACGGATCCTACCGGGT AGCTCCCGATGATATATCAGATG	NUS3063	5' upstream of *cpsJ* + *cpsJ-L-FLAG₃* + 60bp of *cpsI*
P1117	CATTATCCATTAAAAATCAAACGGATCCTACCGGGT AGCTCCCGATGATATATCAGATG		
P1	TAGGATCCGTTTGATTTTTAATGGATAATG	*P-erm* cassette	P-*erm*
P2	GGGCCCCTTTCCTTATGCTTTTG		
P1118	CAAAAGCATAAGGAAAGGGGCCCTTTTCAGATAAAA ATAATGCCATGCAGATCATGCAAC	D39	60bp of *cpsI* + 3' downstre am of *cpsI*
P1119	GACCAACATATCCCGTACCTGCTACTGC		
**For construction of NUS3387 (Δ*cpsI*::*P-erm*)**			
P51	CATTATCCATTAAAAATCAAACGGATCCTACCGGGT AGCTCCCGATGATATATCAGATG	NUS3063	5' upstream of *cpsI* + 60bp of *cpsI*
P1117	CATTATCCATTAAAAATCAAACGGATCCTACCGGGT AGCTCCCGATGATATATCAGATG		
P1	TAGGATCCGTTTGATTTTTAATGGATAATG	*P-erm* cassette	P-*erm*
P2	GGGCCCCTTTCCTTATGCTTTTG		
P1118	CAAAAGCATAAGGAAAGGGGCCCTTTTCAGATAAAA ATAATGCCATGCAGATCATGCAAC	D39	60bp of *cpsI* + *cpsH-L-FLAG₃* + 3' downstre am of *cpsI*
P1119	GACCAACATATCCCGTACCTGCTACTGC		
**For construction of NUS2240, NUS3282 and NUS3298 (Δ*cpsK*::P-*sacB-kan-rpsL⁺*)**			
P1120	GGAACTAGAAAATTGAGTAGAAGATATAATTAC	D39	5' upstream of *cpsK* + 60bp of *cpsK*
P1121	CATTATCCATTAAAAATCAAACGGATCCTAAGACAAT AAAACTGCAATGGATAGACCAAC		
P249	TAGGATCCGTTTGATTTTTAATGGATAATGTTAAGGA TCGATCCGTTTGATTTTTAATGG	P-*sacB-kan-rpsL⁺* cassette	P-*sacB-kan-rpsL⁺*
P250	GGGCCCCTTTCCTTATGCTTTTGGACGTTTAGTACC GTATTTAGAACGG	D39	
P1122	CAAAAGCATAAGGAAAGGGGCCCAACTCATTAGAA GATGTCAAGGAAAAAGTCTATACAC		60bp of *cpsK* + 3' downstre am of *cpsK*
P1123	CAACTTGAACATTTTGCATGCGTTGAACC		
**For construction of NUS3290 (Δ*cpsK*::P-*sacB-kan-rpsL⁺*)**			
P1120	GGAACTAGAAAATTGAGTAGAAGATATAATTAC	D39	5' upstream of *cpsK* +
P1121	CATTATCCATTAAAAATCAAACGGATCCTAAGACAAT AAAACTGCAATGGATAGACCAAC		

			60bp of *cpsK*
P249	TAGGATCCGTTTGATTTTTAATGGATAATGTTAAGGA TCGATCCGTTTGATTTTTAATGG	P-*sacB*-*kan*-*rpsL*$^+$ cassette	P-*sacB*-*kan*-*rpsL*$^+$
P250	GGGCCCCTTTCCTTATGCTTTTGGACGTTTAGTACC GTATTTAGAACGG		
P1122	CAAAAGCATAAGGAAAGGGGCCCAACTCATTAGAA GATGTCAAGGAAAAAGTCTATACAC	NUS3063	*cpsJ-L-FLAG$_3$*+ 60bp of *cpsK* + 3' downstream of *cpsK*
P1123	CAACTTGAACATTTTGCATGCGTTGAACC		
**For construction of NUS2649, NUS3299, NUS3284 and NUS3292 (Δ*cpsP*::P-*sacB-kan-rpsL*$^+$)**			
P1124	AAGAGATGTATTTTCCTATGTATTGCTTATTTTTCAG	D39	5' upstream of *cpsP* + 60bp of *cpsP*
P1125	CATTATCCATTAAAAATCAAACGGATCCTATGGAGG AAGTTCTGTCGCTGAGCGTTCCC		
P249	TAGGATCCGTTTGATTTTTAATGGATAATGTTAAGGA TCGATCCGTTTGATTTTTAATGG	P-*sacB*-*kan*-*rpsL*$^+$ cassette	P-*sacB*-*kan*-*rpsL*$^+$
P250	GGGCCCCTTTCCTTATGCTTTTGGACGTTTAGTACC GTATTTAGAACGG		
P1126	CATTATCCATTAAAAATCAAACGGATCCTATGGAGG AAGTTCTGTCGCTGAGCGTTCCC	D39	60bp of *cpsP* + 3' downstream of *cpsP*
P1127	CTGAAACCTCTGCTTCTGCAATATTTTCCC		
**For construction of NUS2650, NUS3285, NUS3293 and NUS3300 (Δ*cpsL*::P-*sacB-kan-rpsL*$^+$)**			
P1128	GAACGCTTGGTAAGTGCTGCTGAGACATTTG	D39	5' upstream of *cpsL* + 60bp of *cpsL*
P1129	CATTATCCATTAAAAATCAAACGGATCCTAAGCTCGA GTAAGTGGATACAGACGGG		
P249	TAGGATCCGTTTGATTTTTAATGGATAATGTTAAGGA TCGATCCGTTTGATTTTTAATGG	P-*sacB*-*kan*-*rpsL*$^+$ cassette	P-*sacB*-*kan*-*rpsL*$^+$
P250	GGGCCCCTTTCCTTATGCTTTTGGACGTTTAGTACC GTATTTAGAACGG		
P1130	CAAAAGCATAAGGAAAGGGGCCCCAACCTCTTAAG AAGAATGAATACGGACAGTATCTG	D39	60bp of *cpsL* + 3' downstream of *cpsL*
P1131	GATCATAGGCATCCGCAGCTTGTCCC		
**For construction of NUS2652, NUS3327, NUS3377 and NUS3386 (Δ*cpsT*::P-*sacB-kan-rpsL*$^+$)**			
P1424	ATGCCAACGTTAGAAATCTCAC	D39	5' upstream of *cpsT* + 60bp of *cpsT*
P1105	CATTATCCATTAAAAATCAAACGGATCCTAAAATCCT CCATACTTGGCAGGAATCCCC		
P249	TAGGATCCGTTTGATTTTTAATGGATAATGTTAAGGA TCGATCCGTTTGATTTTTAATGG	P-*sacB*-*kan*-*rpsL*$^+$ cassette	P-*sacB*-*kan*-*rpsL*$^+$
P250	GGGCCCCTTTCCTTATGCTTTTGGACGTTTAGTACC GTATTTAGAACGG		
P1106	CAAAAGCATAAGGAAAGGGGCCCACGTGGGAAGAG ATAGTGGTGGATTATGAGGAAGAG	NUS0267	60bp of *cpsT* + Δ*cpsE* +
P65	TCGTCCCACCACTAGATAATAGCC		

			3' downstream of *cpsE*
**For construction of NUS3875, NUS3876 and NUS3877 (Δ*bgaA*::P_{spxB}-*cpsC*-P-*spec*)**			
P86	GTTTGACTGCCGGTGTATCT	NUS1257	5' upstream of *bgaA* + 60bp of *bgaA* + P_{spxB} + *cpsC*
	CACATTATCCATTAAAAATCAAACGGATCCTACTATTTCATTTTGTCCAAATCTGGAACG		
O3710	CGTTCCAGATTTGGACAAAATGAAATAGTAGGATCCGTTTGATTTTTAATGGATAATGTG	NUS0092	60bp of *bgaA* + P-*spec* + 3' downstream of *bgaA*
P6	TGCATGGTTACGATAGTCTTGG		
**For construction of NUS3878 (Δ*bgaA*::P_{spxB}-*cpsT*-P-*spec*)**			
P86	GTTTGACTGCCGGTGTATCT	NUS1257	5' upstream of *bgaA* + 60bp of *bgaA* + P_{spxB}
O3711	GAACCAATGATATAAACTGACTTCTTCATAATGATAACTCTCCTTCAATTTTTTTAAAC		
O3713	ATGAAGAAGTCAGTTTATATC	D39	*cpsT*
O3714	TTACTCACTTTTTCCCCCTTC		
O3712	GAAGAGTTTGAAGGGGGAAAAAGTGAGTAATAGGATCCGTTTGATTTTTAATGGATAAT	NUS0092	60bp of *bgaA* + P-*spec* + 3' downstream of *bgaA*
P6	TGCATGGTTACGATAGTCTTGG		
**For construction of NUS3879 (Δ*bgaA*::P_{spxB}-*cpsI*-P-*spec*)**			
P86	GTTTGACTGCCGGTGTATCT	NUS1257	5' upstream of *bgaA* + 60bp of *bgaA* + P_{spxB}
O3719	GATAAAAAAATATAAGATACTTTTTGTCATAATGATAACTCTCCTTCAATTTTTTTAAAC		
O3721	ATGACAAAAAGTATCTTATATTTTTTATC	D39	*cpsI*
O3722	TCAATTTTCTAGTTCCTTATATAGTTG		
O3720	CATGCAACTATATAAGGAACTAGAAAATTGATAGGATCCGTTTGATTTTTAATGGATAA	NUS0092	60bp of *bgaA* + P-*spec* + 3' downstream of *bgaA*
P6	TGCATGGTTACGATAGTCTTGG		
**For construction of NUS3894 (Δ*bgaA*::P_{spxB}-*cpsG*-P-*spec*)**			
P86	GTTTGACTGCCGGTGTATCT	NUS1257	5' upstream of *bgaA* + 60bp of *bgaA* + P_{spxB}
O3715	GGTATAGATAACTCTTATTCCACCTGTCATAATGATAACTCTCCTTCAATTTTTTTAAAC		
O3717	ATGACAGGTGGAATAAGAGTTATC	D39	*cpsG*
O3718	CTATTTACCGTTTTCAATATATAC		
O3716	CAGGGGTATATATTGAAAACGGTAAATAGTAGGATCCGTTTGATTTTTAATGGATAATG	NUS0092	60bp of *bgaA* + P-

P6	TGCATGGTTACGATAGTCTTGG		*spec* + 3' downstream of *bgaA*
**For construction of NUS1423 (Δ*bgaA*::P*spxB*-*itag-mNEONGreen*)**			
P86	GTTTGACTGCCGGTGTATCT	NUS1368	5' upstream of *bgaA* + 60bp of *bgaA* + P*spxB*-itag
P2073	ATGGATAAAAAAGGTTTGGAAATTTTTTTGGCTTCTG TGAGCGAACTTATTAAAGAG		
P806	ATGGATAAAAAAGGTTTGGAAATTTTTTTGGCTTCTG TTTCCAAAGGGGAAGAGGATAAT	NUS0019	60bp of *bgaA* + mNEONGreen + 3' downstream of *bgaA*
P6	TGCATGGTTACGATAGTCTTGG		
**For construction of NUS1782 (Δ*bgaA*::P*spxB*-*itag-mNEONGreen-H3H4*)**			
P86	GTTTGACTGCCGGTGTATCT	NUS1423	5' upstream of *bgaA* + 60bp of *cpsT* + P*spxB*-*itag-mNEONGreen*
P2941	CAGCTCGCGTACGACGTCTTCCAGTTTATACAGTTC ATCCATC		
P2943	CTGGAAGACGTCGTACGCGAG	CB15	*H3H4*
P2944	AGATACGCTGCACTTCTTCC		
P2942	GTTGAGGAAGAAGTGCAGCGTATCTTTAGCTCTTCT AGGTTTGAG	D39	60bp of *bgaA* + 3' downstream of *bgaA*
P6	TGCATGGTTACGATAGTCTTGG		
**For construction of NUS1836, NUS1890, NUS1891, NUS1996 and NUS2030 (*CEP*::P-*sacB-kan-rpsL*+)**			
P3		D39	5' upstream of *CEP* + 60bp of *CEP*
P2693	CAAACGGATCCTACTCGAGCTTAGCTGACTTCAACC CACTACAG		
P249	TAGGATCCGTTTGATTTTTAATGGATAATGTTAAGGA TCGATCCGTTTGATTTTTAATGG	P-*sacB-kan-rpsL*+ cassette	P-*sacB-kan-rpsL*+
P250	GGGCCCCTTTCCTTATGCTTTTGGACGTTTAGTACC GTATTTAGAACGG		
P2694	TAAACGTCCAAAAGCATAAGGAAAGGGGCCCGTCG CTTTTCATTA	D39	60bp of *CEP* + downstream of *CEP*
P4	TGGGCACGTCATTTCCAATGATTACCTG		
**For construction of NUS1861, NUS2335, NUS2336, NUS2029 and NUS3486 (*CEP*::P*spxB*-*itag-popZ*)**			
P3	ATTTGCCCATCTGGCTGACTAGGAGGAA	NUS1417	5' upstream of *CEP* + 60bp of *CEP* + P*spxB*
P2937	AGAAGCCAAAAAAATTTCCAAACCTTTTTTATCCATA ATGATAACTCTCCTTC		

P2939	ATGGATAAAAAAGGTTTGGAAATTTTTTTGGCTTCTA TGTCCGATCAGTCTCAAG	CB15	*Itag-popZ*
P2940	TTAGGCGCCGCGTCCCCGAG		
P2938	CTCTCGGGGACGCGGCGCCTAAGTCGCTTTTCATT ATAGGTC	NUS0267	60bp of *CEP* + 3' downstream of *CEP*
P4	TGGGCACGTCATTTCCAATGATTACCTG		
**For construction of NUS1783 (Δ*bgaA*::P$_{spxB}$-*cpsC-L-sfgfp-H3H4*)**			
P86	GTTTGACTGCCGGTGTATCT	NUS1257	5' upstream of *bgaA* + 60bp of *bgaA* + P$_{spxB}$-*cpsC*
P2945	CTATCGTGTTTTGTTCTTTCATAATGATAACTCTCCT TCAATT		
P2953	GGTTCCGCTGGCTCCGCTGCTGGTT	NUS0882	*L-sfgfp*
P319	CTACTTATAAAGCTCATCCATGCCGTG		
P2946	GCTCGCGTACGACGTCTTCCAGCTTATAAAGCTCAT CCATGCC	CB15	*H3H4*
P2944	AGATACGCTGCACTTCTTCC		
P2947	GAGGAAGAAGTGCAGCGTATCTTAGTTAGCTCTTCT AGGTTTG	D39	60bp of *bgaA* + + 3' downstream of
P6	TGCATGGTTACGATAGTCTTGG		
**For construction of NUS2333 (*cpsC-H3H4*)**			
P91	GGTGATTGTGGCTATCCTTCA	D39	5' upstream of *cpsC* + *cpsC*
P3178	CAGCAGCTCGCGTACGACGTCTTCCAGTTTCATTTT GTCCAAATC		
P2943	CTGGAAGACGTCGTACGCGAG	CB15	*H3H4*
P2944	AGATACGCTGCACTTCTTCC		
P3179	GTTGAGGAAGAAGTGCAGCGTATCTTAGGAGGAAG TTATGCCAAC	D39	*cpsD* + *cpsE* + 3' downstream of *cpsE*
P65	TCGTCCCACCACTAGATAATAGCC		
**For construction of NUS2334 (*cpsC-H3H4*)**			
P91	GGTGATTGTGGCTATCCTTCA	D39	5' upstream of *cpsC* + *cpsC*
P3178	CAGCAGCTCGCGTACGACGTCTTCCAGTTTCATTTT GTCCAAATC		
P2943	CTGGAAGACGTCGTACGCGAG	CB15	*H3H4*
P2944	AGATACGCTGCACTTCTTCC		
P3179	GTTGAGGAAGAAGTGCAGCGTATCTTAGGAGGAAG TTATGCCAAC	NUS0267	*cpsD* + Δ*cpsE*' + 3' downstream of *cpsE*
P65	TCGTCCCACCACTAGATAATAGCC		
**For construction of NUS4000 (*CEP::P$_{spxB}$-ipopZ-P-spec*)**			
P3	ATTTGCCCATCTGGCTGACTAGGAGGAA	NUS1861	5' upstream of *CEP* + 60bp of *CEP* + *P$_{spxB}$-ipopZ*
O3815	CATTATCCATTAAAAATCAAACGGATCCTATTAGGCG CCGCGTCCCCGAGAGATAC		

O3816	GTATCTCTCGGGGACGCGGCGCCTAATAGGATCCG TTTGATTTTTAATGGATAATG	NUS2905	60bp of *CEP* + 3' downstream of *CEP* + P-spec
P4	TGGGCACGTCATTTCCAATGATTACCTG		

REFERENCE

1. J. A. Lanie, et al., Genome sequence of Avery's virulent serotype 2 strain D39 of *Streptococcus pneumoniae* and comparison with that of unencapsulated laboratory strain R6. *J Bacteriol* **189**, 38–51 (2007).

2. J. Slager, R. Aprianto, J.-W. Veening, Deep genome annotation of the opportunistic human pathogen *Streptococcus pneumoniae* D39. *Nucleic Acids Res* **46**, 9971–9989 (2018).

3. K. M. Kazmierczak, K. J. Wayne, A. Rechtsteiner, M. E. Winkler, Roles of rel_{Spn} in stringent response, global regulation and virulence of serotype 2 *Streptococcus pneumoniae* D39. *Mol Microbiol* **72**, 590–611 (2009).

4. Nakamoto, R., Kwan, J., Chin, J., Ong, H. T., Flores-Kim, J., Midonet, C., VanNieuwenhze, M. S., Guan, X. L., & Sham, L. T, The bacterial tyrosine kinase system CpsBCD governs the length of capsule polymers. *Proc Natl Acad Sci U S A* **118**, 45 (2021).
