# Supplementary material for: The divisome but not the elongasome organizes capsule synthesis in Streptococcus pneumoniae
Source: Nat Commun. 2023 Jun 1;14:3170. doi: 10.1038/s41467-023-38904-9 (PMC10233549; doi:10.1038/s41467-023-38904-9)
Supplement: Supplementary file 3 — Reporting Summary [file 41467_2023_38904_MOESM3_ESM.pdf]

nature portfolio

Corresponding author(s): Lok-To Sham

Last updated by author(s): May 11, 2023

# Reporting Summary

Nature Portfolio wishes to improve the reproducibility of the work that we publish. This form provides structure for consistency and transparency in reporting. For further information on Nature Portfolio policies, see our Editorial Policies and the Editorial Policy Checklist.

## Statistics

For all statistical analyses, confirm that the following items are present in the figure legend, table legend, main text, or Methods section.

| n/a | Confirmed | |
|---|---|---|
| ☐ | ☒ | The exact sample size (*n*) for each experimental group/condition, given as a discrete number and unit of measurement |
| ☐ | ☒ | A statement on whether measurements were taken from distinct samples or whether the same sample was measured repeatedly |
| ☐ | ☒ | The statistical test(s) used AND whether they are one- or two-sided<br>*Only common tests should be described solely by name; describe more complex techniques in the Methods section.* |
| ☒ | ☐ | A description of all covariates tested |
| ☒ | ☐ | A description of any assumptions or corrections, such as tests of normality and adjustment for multiple comparisons |
| ☐ | ☒ | A full description of the statistical parameters including central tendency (e.g. means) or other basic estimates (e.g. regression coefficient) AND variation (e.g. standard deviation) or associated estimates of uncertainty (e.g. confidence intervals) |
| ☐ | ☒ | For null hypothesis testing, the test statistic (e.g. *F*, *t*, *r*) with confidence intervals, effect sizes, degrees of freedom and *P* value noted<br>*Give P values as exact values whenever suitable.* |
| ☒ | ☐ | For Bayesian analysis, information on the choice of priors and Markov chain Monte Carlo settings |
| ☒ | ☐ | For hierarchical and complex designs, identification of the appropriate level for tests and full reporting of outcomes |
| ☒ | ☐ | Estimates of effect sizes (e.g. Cohen's *d*, Pearson's *r*), indicating how they were calculated |

*Our web collection on statistics for biologists contains articles on many of the points above.*

## Software and code

Policy information about availability of computer code

| | |
|---|---|
| Data collection | The following softwareas were used for data collection:<br>ImageJ (https://imagej.nih.gov/ij/download.html)<br>MicrobeJ (v. 5.13l; https://www.microbej.com/download-2/)<br>Oufti (https://www.oufti.org/). |
| Data analysis | GraphPad Prism (v. 9.5.1; https://www.graphpad.com/features) was used to perform statistical analyses and draw figures<br>FlowJo (v10.8.1) was used to analyze flow cytometry data |

For manuscripts utilizing custom algorithms or software that are central to the research but not yet described in published literature, software must be made available to editors and reviewers. We strongly encourage code deposition in a community repository (e.g. GitHub). See the Nature Portfolio guidelines for submitting code & software for further information.

## Data

Policy information about availability of data

All manuscripts must include a data availability statement. This statement should provide the following information, where applicable:

- Accession codes, unique identifiers, or web links for publicly available datasets
- A description of any restrictions on data availability
- For clinical datasets or third party data, please ensure that the statement adheres to our policy

All data generated or analysed during this study are included in this published article (and its supplementary information files). Source data are provided with this paper, or from the corresponding author upon request.

## Human research participants

Policy information about studies involving human research participants and Sex and Gender in Research.

| | |
|---|---|
| Reporting on sex and gender | N/A |
| Population characteristics | N/A |
| Recruitment | N/A |
| Ethics oversight | N/A |

Note that full information on the approval of the study protocol must also be provided in the manuscript.

# Field-specific reporting

Please select the one below that is the best fit for your research. If you are not sure, read the appropriate sections before making your selection.

[x] Life sciences [ ] Behavioural & social sciences [ ] Ecological, evolutionary & environmental sciences

For a reference copy of the document with all sections, see nature.com/documents/nr-reporting-summary-flat.pdf

# Life sciences study design

All studies must disclose on these points even when the disclosure is negative.

| | |
|---|---|
| Sample size | No sample size calculation was performed. Samples sizes were chosen based on the convention in the field following the generally accepted precedence of statistical analyses for mechanistic studies (e.g. PMID: 34475211, 30651563, 34732571, etc.) |
| Data exclusions | No data was excluded in this study. |
| Replication | The number of independent biological replicates were indicated in the paper for each experiment. Results produced were similar. |
| Randomization | Randomization is not applicable for this study. |
| Blinding | Blinding is not applicable for this study. |

# Reporting for specific materials, systems and methods

We require information from authors about some types of materials, experimental systems and methods used in many studies. Here, indicate whether each material, system or method listed is relevant to your study. If you are not sure if a list item applies to your research, read the appropriate section before selecting a response.

## Materials & experimental systems

| n/a | Involved in the study |
|---|---|
| | ☒ Antibodies |
| ☒ | ☐ Eukaryotic cell lines |
| ☒ | ☐ Palaeontology and archaeology |
| ☒ | ☐ Animals and other organisms |
| ☒ | ☐ Clinical data |
| ☒ | ☐ Dual use research of concern |

## Methods

| n/a | Involved in the study |
|---|---|
| ☒ | ☐ ChIP-seq |
| ☒ | ☐ Flow cytometry |
| ☒ | ☐ MRI-based neuroimaging |

# Antibodies

Antibodies used

Anti-FLAG polyclonal antibody (Sigma F7425);
Alexa Fluor 647 conjugated anti-rabbit IgG (Thermo scientific A-31573);
Anti-serotype 2 CPS antiserum (SSI 16745);
Anti-GFP antibodies (Abcam ab1218);
Goat anti-rabbit HRP antibodies (Thermo Fisher A16110);
Goat anti-mouse HRP antibodies (Thermo Fisher G-21040);
Goat anti-C3b antibody (Calbiochem 204869);
Alexa Fluor 594 conjugated donkey anti-rabbit IgG (Invitrogen A-21207);
Alexa Fluor 488 conjugated donkey anti-goat antibody (Invitrogen A-11055)

Validation

All antibodies used in this study are commercially available. They have been validated by the manufacturers for their use in immunoblotting, cell sorting experiments, and/or immunofluorescence microscopy. The descriptions of their validation are available on their websites, which are listed below:

Anti-FLAG polyclonal antibody (Sigma F7425): https://www.sigmaaldrich.com/SG/en/product/sigma/f7425
Alexa Fluor 647 conjugated anti-rabbit IgG (Thermo scientific A-31573): https://www.thermofisher.com/antibody/product/Donkey-anti-Rabbit-IgG-H-L-Highly-Cross-Adsorbed-Secondary-Antibody-Polyclonal/A-31573
Anti-serotype 2 CPS antiserum (SSI 16745): https://shop.ssidiagnostica.com/pneumokok-type-2-serum-1-ml.html
Anti-GFP antibodies (Abcam ab1218): https://www.abcam.com/products/primary-antibodies/gfp-antibody-9f9f9-ab1218.html
Goat anti-rabbit HRP antibodies (Thermo Fisher A16110): https://www.thermofisher.com/antibody/product/Goat-anti-Rabbit-IgG-H-L-Highly-Cross-Adsorbed-Secondary-Antibody-Polyclonal/A16110
Goat anti-mouse HRP antibodies (Thermo Fisher G-21040): https://www.thermofisher.com/antibody/product/Goat-anti-Mouse-IgG-H-L-Cross-Adsorbed-Secondary-Antibody-Polyclonal/G-21040
Goat anti-C3b antibody (Calbiochem 204869): https://www.merckmillipore.com/SG/en/product/Anti-Complement-3-Goat-pAb,EMD_BIO-204869
Alexa Fluor 594 conjugated donkey anti-rabbit IgG (Invitrogen A-21207): https://www.thermofisher.com/antibody/product/Donkey-anti-Rabbit-IgG-H-L-Highly-Cross-Adsorbed-Secondary-Antibody-Polyclonal/A-21207
Alexa Fluor 488 conjugated donkey anti-goat antibody (Invitrogen A-11055): https://www.thermofisher.com/antibody/product/Donkey-anti-Goat-IgG-H-L-Cross-Adsorbed-Secondary-Antibody-Polyclonal/A-11055
